# Toxicological assessment of pristine and degraded forms of graphene functionalized with MnOx nanoparticles using human in vitro models representing different exposure routes

**DOI:** 10.1038/s41598-023-38993-y

**Published:** 2023-07-22

**Authors:** Natalia Fernández-Pampín, Juan José González Plaza, Alejandra García-Gómez, Elisa Peña, Sebastiano Garroni, Matteo Poddighe, Carlos Rumbo, Rocío Barros, Sonia Martel-Martín, Santiago Aparicio, Juan Antonio Tamayo-Ramos

**Affiliations:** 1 International Research Centre in Critical Raw Materials-ICCRAM, University of Burgos, Plaza Misael Bañuelos s/n, 09001 Burgos, Spain; 2 Gnanomat, C/Faraday 7, 28049 Madrid, Spain; 3 Department of Chemical, Physics, Mathematics and Natural Science, University of Sassari, Via Vienna 2, 07100 Sassari, Italy; 4 Laboratory of Materials Science and Nanotechnology (LMNT), Department of Chemical, Physics, Mathematics and Natural Science, CR-INSTM, University of Sassari, Via Vienna, 2, 07100 Sassari, Italy

**Keywords:** Graphene, Nanotoxicology

## Abstract

The development of novel advanced nanomaterials (NMs) with outstanding characteristics for their use in distinct applications needs to be accompanied by the generation of knowledge on their potential toxicological impact, in particular, that derived from different occupational risk exposure routes, such as inhalation, ingestion, and skin contact. The harmful effects of novel graphene-metal oxide composites on human health are not well understood, many toxicological properties have not been investigated yet. The present study has evaluated several toxicological effects associated with graphene decorated with manganese oxide nanoparticles (GNA15), in a comparative assessment with those induced by simple graphene (G2), on human models representing inhalation (A549 cell line), ingestion (HT29 cell line) and dermal routes (3D reconstructed skin). Pristine and degraded forms of these NMs were included in the study, showing to have different physicochemical and toxicological properties. The degraded version of GNA15 (GNA15d) and G2 (G2d) exhibited clear structural differences with their pristine counterparts, as well as a higher release of metal ions. The viability of respiratory and gastrointestinal models was reduced in a dose-dependent manner in the presence of both GNA15 and G2 pristine and degraded forms. Besides this, all NMs induced the production of reactive oxygen species (ROS) in both models. However, the degraded forms showed to induce a higher cytotoxicity effect. In addition, we found that none of the materials produced irritant effects on 3D reconstructed skin when present in aqueous suspensions. These results provide novel insights into the potentially harmful effects of novel multicomponent NMs in a comprehensive manner. Furthermore, the integrity of the NMs can play a role in their toxicity, which can vary depending on their composition and the exposure route.

## Introduction

Forging ground-breaking scientific research requires the fuel of human dreams, which in the frontiers of imagination and science are introduced as scientific hypotheses. Such were the early concepts of the “nano” presented by Feynman^1^. Nanomaterials (NMs) range from 1 to 100 nm, for at least 50% of particles^2^, and present a myriad of applications, generating important revenues and high-quality employment^3^. Some of these applications include the way we produce and store energy^4^, construction materials that can self-clean^5^ decreasing CO_2_ footprint, improvement of soil properties for more sustainable agricultural practices^6^, or providing opportunities for new treatments^7^. A new generation of nanoparticles (NPs) is based on the hybridisation of carbon-derived NMs with transition metal oxides (TMO): titanium dioxide^8^, manganese oxide^9^, iron oxide^10^, or zinc oxide^11^. These prevent the aggregation of individual graphene sheets caused by strong van der Waals interaction^12^, and present improved catalytic, magnetic and optoelectronic properties^13,14^. In particular, Mn-containing complexes loaded into carbon matrices have interesting pseudocapacitance properties^15^, and are supercapacitor materials^16^. Notwithstanding, the harmful effects of these novel graphene-metal oxide composites on human health and the environment must be evaluated, not only on their pristine forms but also on their degradation products, attending as well to the mixture toxicity concept. 2D NMs present an enormous functional surface area^17^, where a range of biomolecules (e.g. enzymes) could be mobilised impairing their regular function within the cell. Also, their particular shape can physically damage cellular membranes^18^. Therefore, it is crucial to know the possible relationship between the different size of graphene and the associated toxic effects on various biological systems such as human cells, zebrafish, and bacteria to ensure their safe utility^19^. Additionally, along their life cycle, they can suffer changes in their structure and composition, which has been reported to alter their toxicological properties^20^.

Scientific efforts have been done to generate knowledge on graphene hazard in many in vivo and in vitro models, to understand its toxic effects in different human cell models and organs^21^, as well as in environmental systems^22,23^. Also, the toxicity of metal oxide nanoparticles has been determined in human^24^, and environmental models^25^. However, the toxicological properties of graphene-metal oxide composites are far less understood. Although some reports have been recently published describing the determination of the combined effects of graphene and metal oxide nanoparticles, the availability of studies is still scarce, focusing on a very limited number of samples and biological models, always in pristine samples^26^. In particular, the toxicological properties of manganese oxide-functionalized graphene have not been assessed yet, despite its great functionality potential. On the other hand, graphite materials are released into the environment, producing toxic effects on flora and fauna^27–29^. As far as we know, there is no report on the impact of the degradation of graphene caused by physical phenomena on toxic effects.

In the present study, we have evaluated and compared the toxicological effects of two pristine NMs of different compositional complexity, and their degraded forms. For that purpose, we have employed two different human cell lines representing respiratory and gastrointestinal exposure routes, as well as a three-dimensional human skin model to determine the irritation potential of all evaluated samples. One of these materials was a classical graphene formulation (G2), and the other one was a functionalized form of G2 (GNA15), carrying immobilised MnOx nanoparticles (MnOx-NPs) on its surface, developed to enhance its specific capacitance (pseudocapacitance). Due to its recent development, the toxicological effects of this multicomponent NM are poorly understood. To obtain insights into the potential toxicity of aged forms as well, we have obtained degraded versions of G2 and GNA15 to understand whether physicochemical changes can alter their original toxicity.

We aimed to answer questions related to the potential synergistic toxicity of combined graphene and Mn oxide NMs, as well as to mixture toxicity that might occur. For this purpose, the effects of MnOx-NPs functionalized graphene, together with that of their components by separate, graphene and MnOx-NPs, were analysed and compared, considering the results obtained in a previous work published by our group regarding the toxicity of the latter^30^. Our comparative toxicological assessment through in vitro survival and ROS assays has considered respiratory, gastrointestinal and dermal exposure routes, and brings new light on the potential toxicity of pristine and degraded forms of novel advanced NMs.

## Results

### NMs physicochemical characterisation

MnOx-NPs functionalized graphene (GNA15) was synthesised by the company Gnanomat, employing MnO_x_-NPs and graphene (G2), through a nanotechnological patented process (Patent number ES2678419A1). To determine the NM composition and its thermal stability TGA analysis was carried out. We found weight losses starting at 300 °C due to the elimination of surface and occluded water, organic compounds, and trace amounts of oxygen (Supplementary Fig. S1). Beyond 600 °C the weight loss was detected in GNA15 NM, which corresponds to the decomposition of carbon material in air, indicating a final composite MnOx-NPs content of 53%. However, a mass gain was observed above 600 °C in the case of GNA15d NM. According to R. Amankwah et al.^31^ the increased mass could be due to the oxidation of manganese oxide (Supplementary Fig. S1).

**Figure 1:**
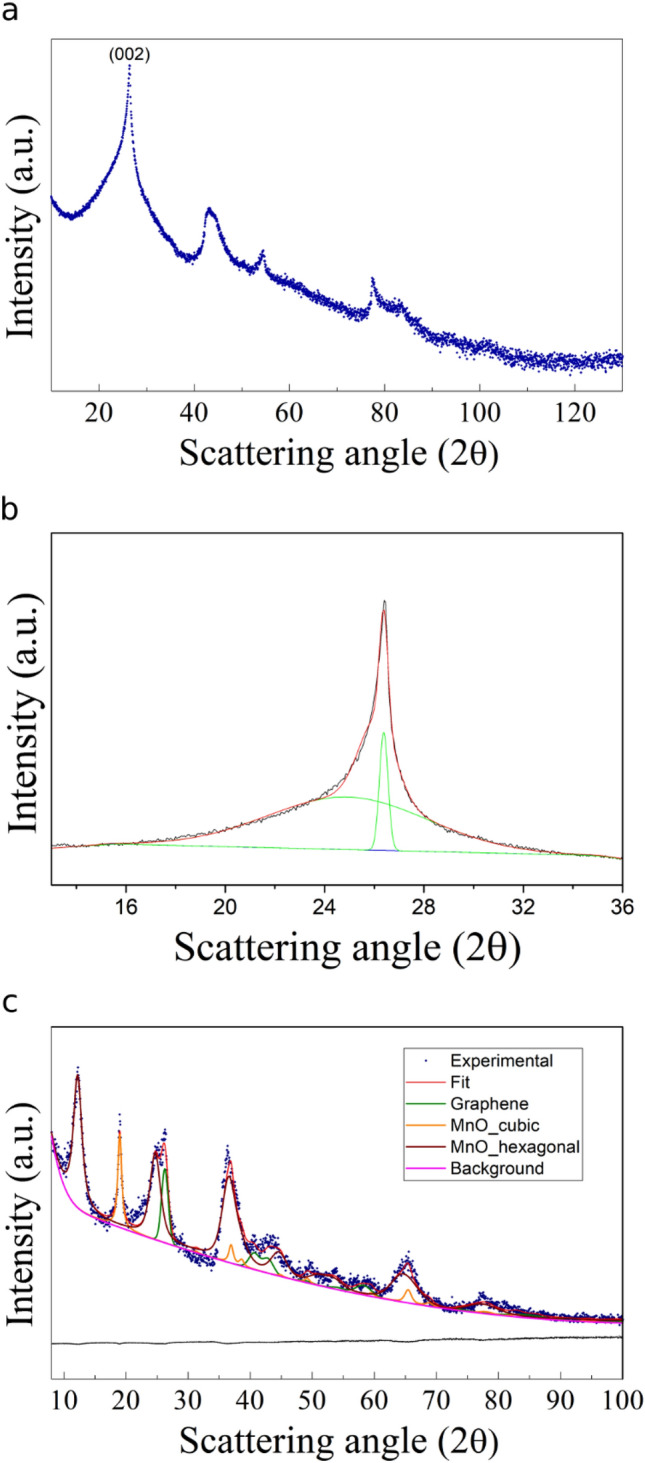
XRD characterisation of G2 and GNA15. (**a**) XRD pattern of G2. (**b**) Focus on *002* peak of G2. Experimental: black line; Best-Fit: red line; Interpolation by two gaussian functions: green lines. (**c**) GNA15 XRD profile.

GNA15 and G2 were also characterised through X-ray diffraction analysis to obtain insights into their crystalline structures. The XRD pattern of the MnOx-NPs employed to obtain GNA15 showed two crystalline phases, tetragonal Mn_3_O_4_ (SG: *I41/amd:1*) and cubic Mn_2_O_3_ (SG: *Ia-3*) (Fig. S2). The quantitative evaluation suggests that the tetragonal manganese oxide is approximately 67.8% (wt.) with an average crystallite size of 102 nm. The microstructural and structural parameters, estimated by Rietveld analysis, are reported in Supplementary Table S1. The XRD pattern of G2 presents a significant reduction in intensities of the *002*-reflection compared with pure graphite^32^, revealing a strong and sharp diffraction peak at around 26.52° (*002*) due to its high degree of crystallinity (Fig. 1a). The location of G2 decreases at 26.38° due to an interlayer *d*_022_ spacing of 3.35 Å, comparable with that of bulk graphite (3.34 Å). A shoulder at the lower angle of 24.92° (*d* = *3.57* Å*)* can be also detected and it was related to the few-layer graphene^33^. The two peaks were interpolated by Gauss functions (Fig. 1b). The Debye–Scherrer equation was applied, indicating that G2 corresponds to a mixture of graphene sheets with a different number of layers, estimable from 60 to 3. The XRD profile for GNA15 (Fig. 1c) revealed three main nanocrystalline phases: graphene (SG *P63/mmc*), and a mixture of non-stoichiometric MnOx species characterised by a cubic (SG *Fd-3m:2*) and hexagonal (SG *P63/mmc*) crystalline habits. The *002* peak of graphene is shifted at lower angles, 26.22°, which could be related to an interlayer distance increase upon the confinement of manganese oxide nanoparticles. The broad peak evidenced in Fig. 1b cannot be revealed because it overlapped with MnOx reflection. Structural and microstructural parameters are reported in Supplementary Table S2.

**Figure 2:**
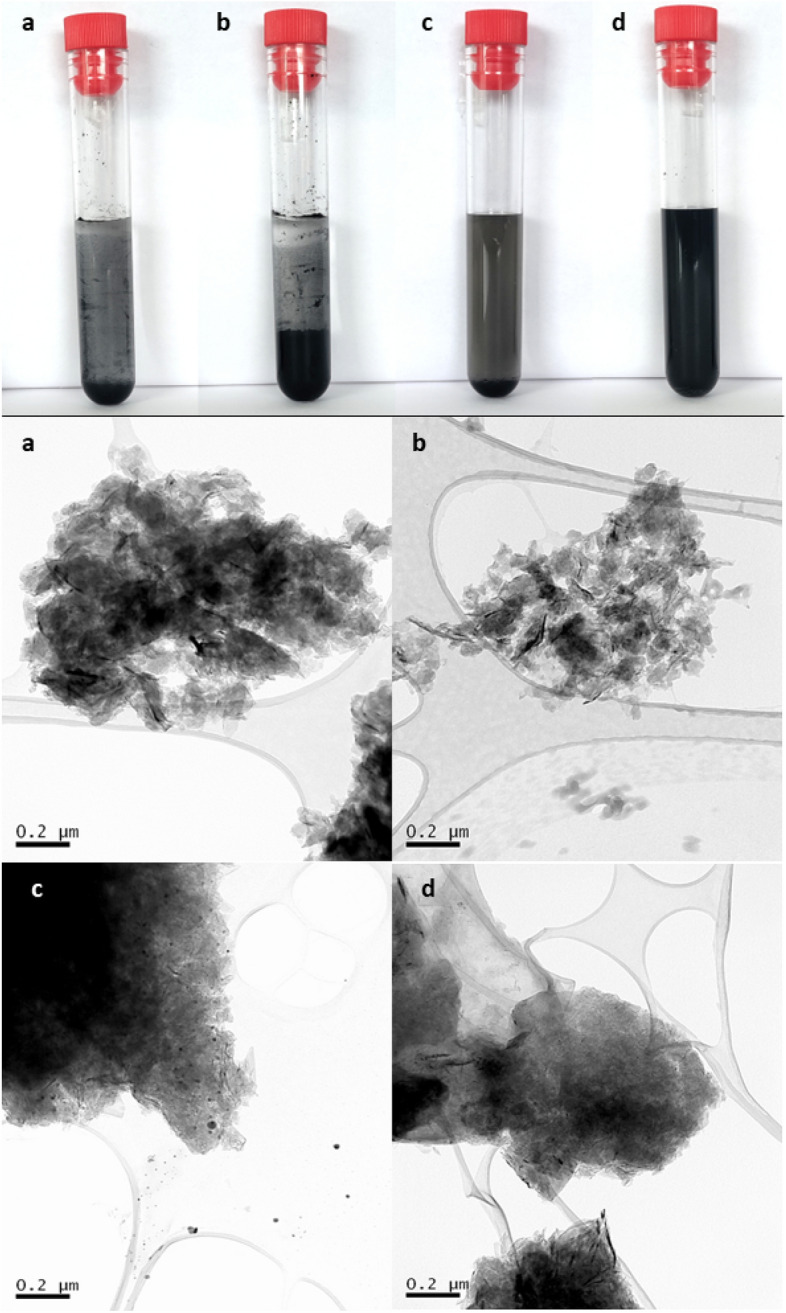
Images of the NMs used in this study. Upper panel: pictures of the NMs in suspension. Lower four panels: TEM analysis of pristine and ultrasonicated materials. Key: Pristine G2 (**a**), ultrasonicated G2 (**b**), pristine GNA15 (**c**), ultrasonicated GNA15 (**d**).

To be able to study structural, compositional and toxicological features of degraded forms of G2 (G2d) and GNA15 (GNA15d), liquid suspensions of both NMs were subjected to a heavy ultrasound treatment (see “Material and methods” section). Differences between pristine and degraded forms were assessed through transmission electron microscopy (TEM), Raman and ICP-MS. Visual observation of the NMs suspensions already indicates different sedimentation rates between those containing pristine and degraded forms (Fig. 2). As shown in the upper panel of Fig. 2, the NMs tend to precipitate, observing a higher precipitate in the G2 and G2d solutions. Different sedimentation rates could be related to the particle size. These visual perceptions were confirmed by TEM and DLS analyses. TEM analysis also showed sheet-like morphology differences between both types of pristine and degraded NMs (Fig. 2). Specially in the case of degraded nanomaterials, it can observe higher transparent areas in the periphery of the sheets. This could be suggesting a reduction of the layer thickness, which could be related to the degradation process (Fig. 2). Moreover, it was observed in the center of the particle’s areas with high electron density, particularly in G2 and GNA15d. It could indicate the presence of several layers in G2. However, in the case of GNA15d it could correspond with remnants generated in the degradation process. Moreover, small particles were visible in G2d, they could have been produced during the degradation process. Particle size and stability were analysed as well through dynamic light scattering (DLS) and ζ-potential, in ultrapure water and cell culture media (DMEM + 1% FBS) suspensions, at a concentration of 50 mg mL^−1^. Therefore, based on signal intensity (Figs. S3 and S4) the particle size is the particle size for the NMs resuspended in water was: 432 ± 30 nm for G2, 425 ± 28 nm for G2d, 224 ± 16. nm for GNA15 and 334 ± 8 nm for GNA15d, while their respective ζ-potential was—(20 ± 1) for G2,—(31 ± 1) for G2d,—(34 ± 1) for GNA15,—(35 ± 1) for GNA15d. The obtained ζ-potential values were similar for all the NMs, showing that all of them are stable in solution. Comparing the particle size, the DLS analysis indicated that GNA15 and GN15d are smaller than G2 and G2, therefore they tend to precipitate less. When the NMs were dissolved in the culture medium, all the NMs, except G2, were stable to perform the DLS measurements. The values obtained for culture medium solutions were higher than in the water solutions in the case of G2d and GNA15, 843.7 ± 28.5 nm for G2d, 509 ± 46.1 nm for GNA15. However, GNA15d showed lower values in the culture medium (250.3 ± 6.7 nm). This could be due to the presence of proteins in the medium, which could help to stabilize the particles, avoiding this way their aggregation. Regarding the ζ-potential measurements in culture medium solutions, they could not be carried out due to the high conductivity and colourations of the cell culture medium. Moreover, DLS also provides the polydispersity index (PDI), which indicates the width of the particle size distribution. A value of PDI < 0.1 indicates that the sample is monodisperse while PDI values between 0.1 < PDI < 0.2 indicate that the sample would have a narrow particle size distribution. The spectrum of the DLS (Figs. S3 and S4) show that all the particle population, except GNA15 in medium, follow a Gaussian distribution, this indicates that the samples are monodisperse. Thus, PDI in water was (0.45 ± 0.04) for G2, (0.48 ± 0.06) for G2d, (0.37 ± 0.07) for GNA15 and (0.52 ± 0.02) for GNA15d. In the cell culture medium, PDI was (0.42 ± 0.02) for G2d, (0.64 ± 0.06) for GNA15 and (0.30 ± 0.04) for GNA15d. PDI values between 0.2 < PDI < 0.5 indicate that the sample has a wide particle size distribution according to the results reported by J. Lohrke et al.^34^.

**Figure 3:**
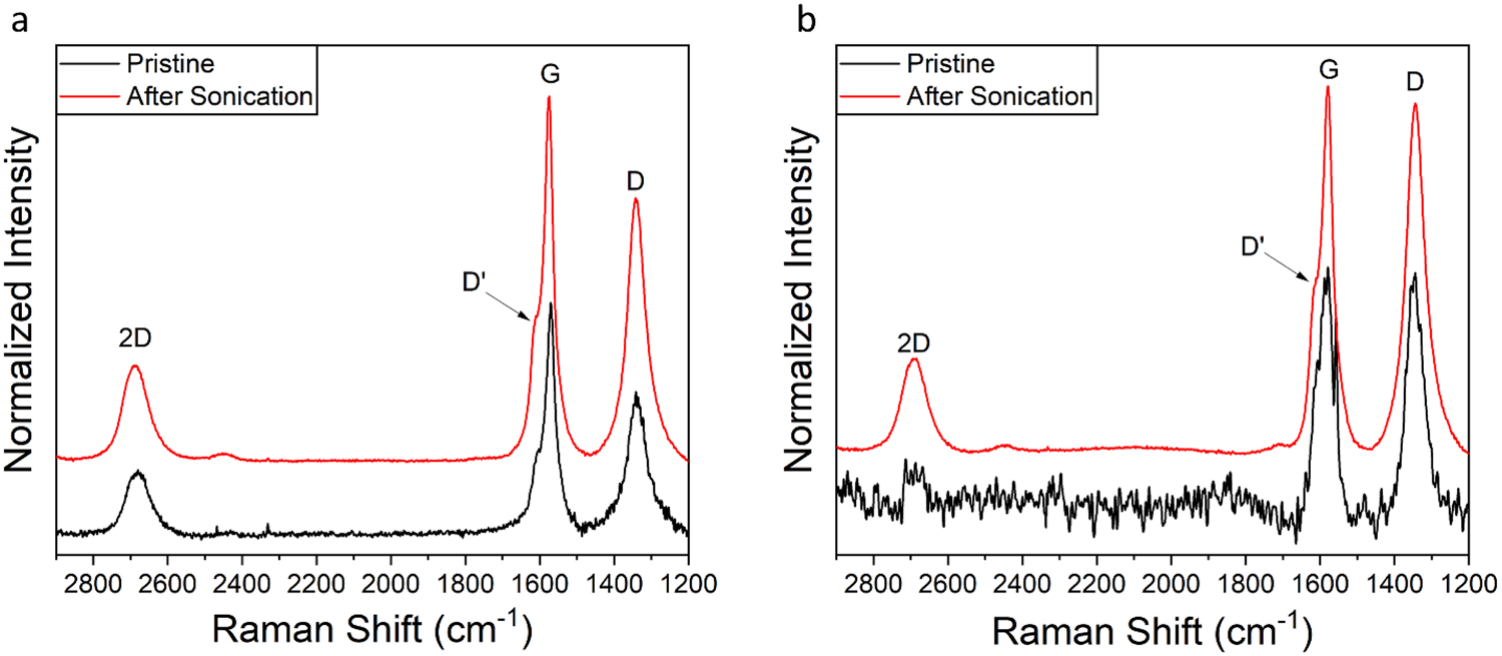
Raman analysis of pristine and ultrasonicated G2 (**a**) and GNA15 (**b**). Black lines: pristine form; red lines: ultrasonicated form.

Raman analysis also shows clear differences between pristine and degraded forms (Fig. 3). As can be observed in Fig. 3, G2d and GNA15d display more intense and narrower peaks. The D and D′ bands are indicative of material defects, e.g. effect of the presence of sp^3^ carbons within the structure. We observe a characteristic graphene band (G). The 2D band is the second order of the D band, and it is used to measure the graphene layer thickness. The degradation treatment increases the intensity of 2D for both G2 (Fig. 3a) and GNA15 (Fig. 3b), which becomes more symmetric and narrower. This observation indicates the presence of more exfoliated materials when compared to the pristine ones. In addition to the structural changes observed in the degraded materials, the study of the released metal ions in water and culture medium was analysed by ICP-MS on the supernatant of pristine and degraded NMs suspensions. The obtained results showed a significantly higher concentration of metal ions in supernatants of the degraded forms. This is particularly remarkable in the case of GNA15, where the Mn concentration present in the water solution of the degraded form (5.69 ppm) was found to be almost 60 times higher than that of the pristine one (0,09 ppm) (Supplementary Table S3). Regarding the Mn ions released into the culture medium, as well as in water solutions, the presence of Mn ions in the supernatant of GNA15 was major (10.25 ppb) than in the G2 supernatant (3.53 ppb), whereas the Mn ions concentration in the supernatant of the degraded forms was higher than in pristine forms, GNA15d (93.13 ppb) and G2d (2.87 ppb). These results indicate that the degradation process favours the ions release.

### Toxicity evaluation of pristine and degraded G2 and GNA15 in the human alveolar carcinoma epithelial cells A549

The cytotoxic effect induced in the A549 cell line upon exposure to G2, GNA15 and their degraded forms was analysed by MTT assay and ROS determination.

**Figure 4:**
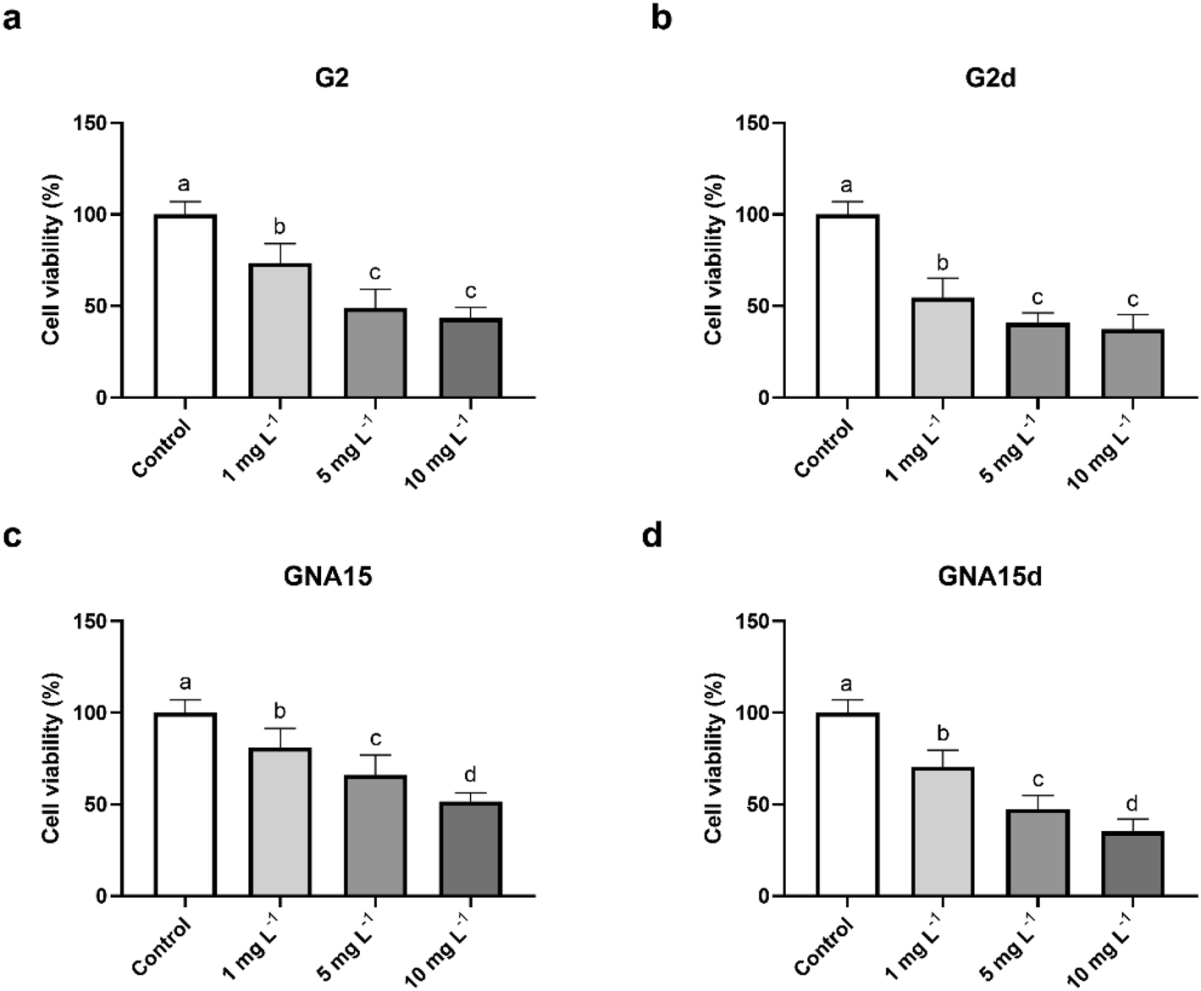
Viability of A549 cells exposed to different concentrations of G2 (**a**), G2d (**b**), GNA15 (**c**) and GNA15d (**d**) for 24 h. Results are expressed as % of control (non-exposed cells). Data represented the mean (± standard deviation, SD) of two independent experiments (where in total N = 6). Differences were established using one-way ANOVA followed by multiple comparisons test (Tukey test) and considered significant when *p* ≤ 0.05. Different letters indicate statistically significant differences between treatments.

The viability assay was performed in cells exposed to the NMs under study at different concentrations (10, 5 and 1 mg L^−1^), for 24 h. On one hand, the obtained results showed a statistically significant viability reduction in the exposed cells in comparison with the control (non-exposed cells) after 24 h of exposure (Fig. 4 and Supplementary Table S4). Moreover, this effect was higher and statically significant after the treatment with G2 at 5 and 10 mg L^−1^ (Supplementary Tables S4 and S5). On the other hand, when analysing how the degradation process affects A549 cells, we observed that degraded forms produce a stronger decline in cell viability than the pristine materials, mainly at higher concentrations (Fig. 4 and Supplementary Table S4), being statistically significant in the case of GNA15 vs GNA15d, at 5 and 10 mg L^−1^. However, there are no statically significant differences in the cytotoxicity between degraded NMs (Supplementary Table S5).

**Figure 5:**
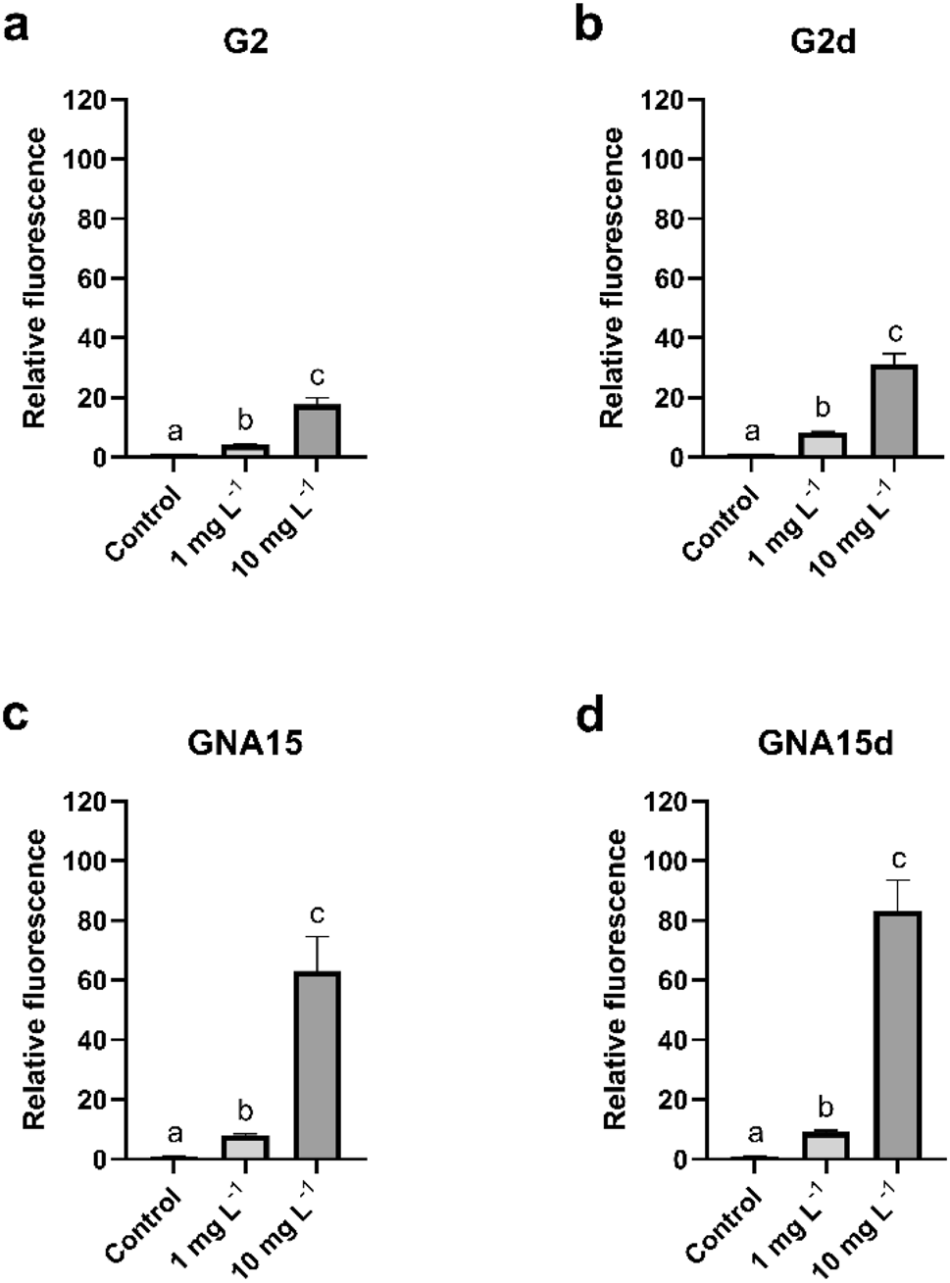
ROS of A549 cells exposed to different concentrations of G2 (**a**), G2d (**b**), GNA15 (**c**) and GNA15d (**d**) for 60 min. Results are expressed as % of control (non-exposed cells). Data represented the mean (± standard deviation, SD) of two independent experiments (where in total N = 6). Differences were established using one-way ANOVA followed by multiple comparisons test (Tukey test) and considered significant when *p* ≤ 0.05. Different letters indicate statistically significant differences between treatments.

The levels of ROS in A549 cells were determined using the DCFH-DA reagent. The cells were exposed to 1 and 10 mg L^−1^ for 1 h, and then, ROS levels were measured at different times (0, 30 and 60 min) (Supplementary Figs. S5 and S6). The obtained results indicate that ROS production is dependent on the exposure time and the NMs concentration. As can be observed in Supplementary Figs. S3 and S4, after the exposure to the tested materials, the increase in the generation of ROS levels was statistically significant concerning the non-exposed cells, showing the highest values at 60 min and 10 mg L^−1^. The fluorescence ratios between A549 cells' ROS response to different treatments and the statistically significant differences between conditions are represented in Supplementary Tables S6 and S7 respectively. As shown in Fig. 5, at 60 min of exposure GNA15 and GNA15d generated an increase in ROS levels higher than G2 and G2d. Moreover, when analysing the effect of the degraded materials on the A549 cells' ROS levels, the obtained results showed a statically significant increase concerning the pristine materials (Supplementary Table S7), being higher in the case of GNA15d (Supplementary Table S6). These results are in line with the cell viability assays, where the degraded forms presented a major cytotoxic effect on cell viability than the pristine materials.

### Toxicity evaluation of pristine and degraded G2 and GNA15 in the human colon cancer cell line HT29

**Figure 6:**
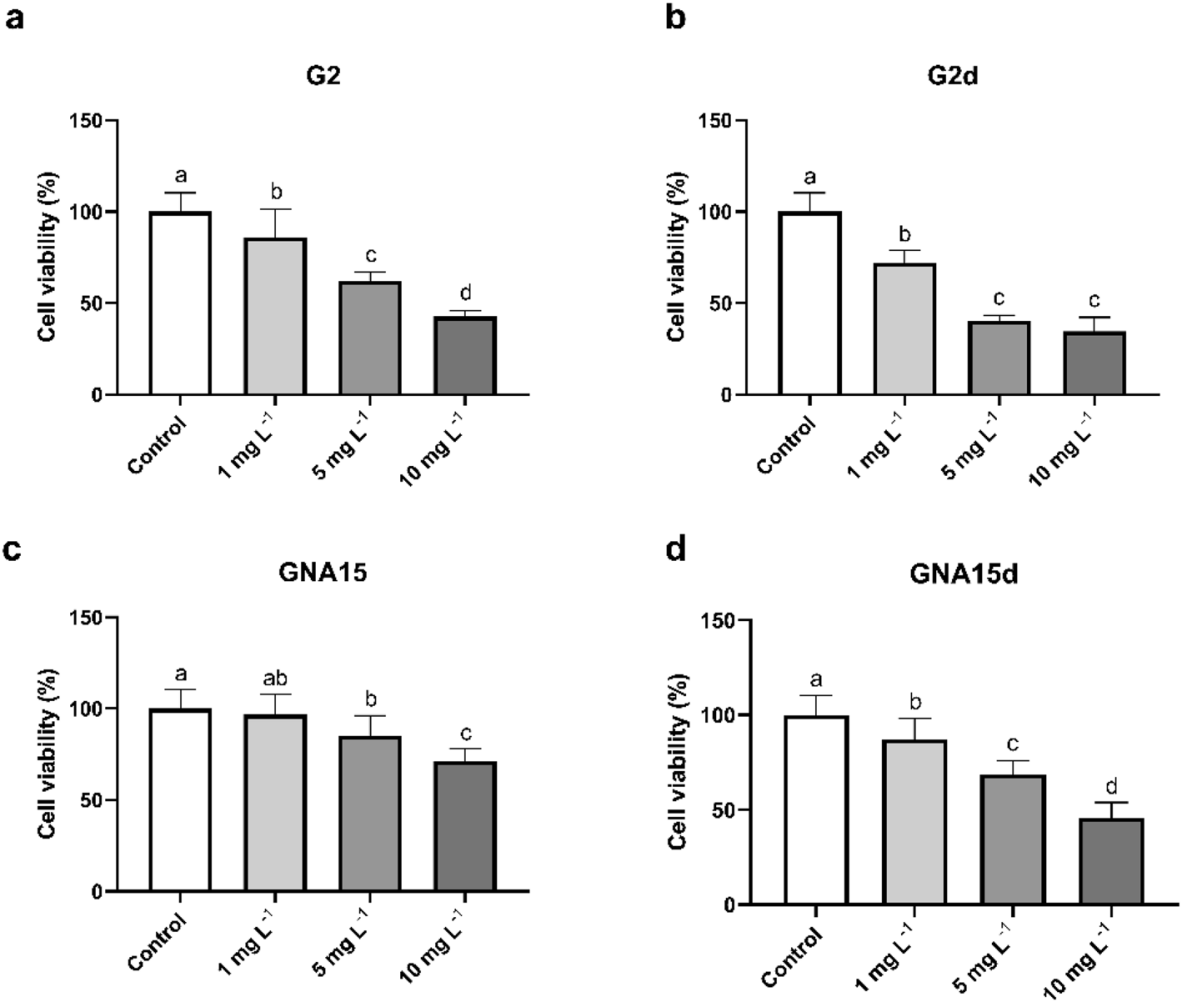
Viability of HT-29 cells exposed to different concentrations of G2 (**a**), G2d (**b**), G2d (**c**) and GNA15 (**d**) for 24 h. Results are expressed as % of control (untreated cells). Data represented the mean (± standard deviation, SD) of two independent experiments (where in total N = 6). Differences were established using a one-way ANOVA followed by multiple comparisons test (Tukey test) and considered significant when* p* ≤ 0.05. Different letters indicate significant differences between treatments.

To evaluate the toxicological impact of G2, G2d, GNA15 and GNA15d in the gastrointestinal model HT29, MTT and ROS assays were performed, employing the same experimental design as that used for A549 cells. Regarding the viability assay, HT29 cells were exposed to different concentrations (1, 5 and 10 mg L^−1^) of the tested materials for 24 h. The results obtained (Fig. 6) show that the tested materials reduce the cell viability at the employed concentrations, except in the case of GNA15 at 1 mg L^−1^, where the values of the cell viability were similar to the control. In addition, when comparing the effects of the pristine materials on the cell viability, it can be observed that G2 produces a stronger reduction in HT-29 cell viability than GNA15. Also, the degraded materials induce a higher loss of cell viability than the pristine materials. In Supplementary Table S8, the different fluorescence ratios between HT-29 cells' response to tested materials are shown, allowing us to compare the impact that different NMs (e.g. G2d vs GNA15d) exert in their viability. Differences are statistically significant in most of the studied conditions, except in most of the cases where the lowest concentration of the NMs was employed (Supplementary Table S9). In addition, as in the case of A549 cells, the reduction of cell viability is dependent on the concentration used.

ROS levels were determined after the exposure of HT-29 cells to G2, GNA15 and their respective degraded forms at different times (0, 30 and 60 min) and concentrations (1, and 10 mg L^−1^) (Supplementary Figs. S7 and S8). The obtained results show that ROS production is dependent on time and concentration. The differences between all tested conditions are statistically significant, except for G2 at time 0 and 1 mg L^−1^ (Supplementary Fig. S7).

**Figure 7:**
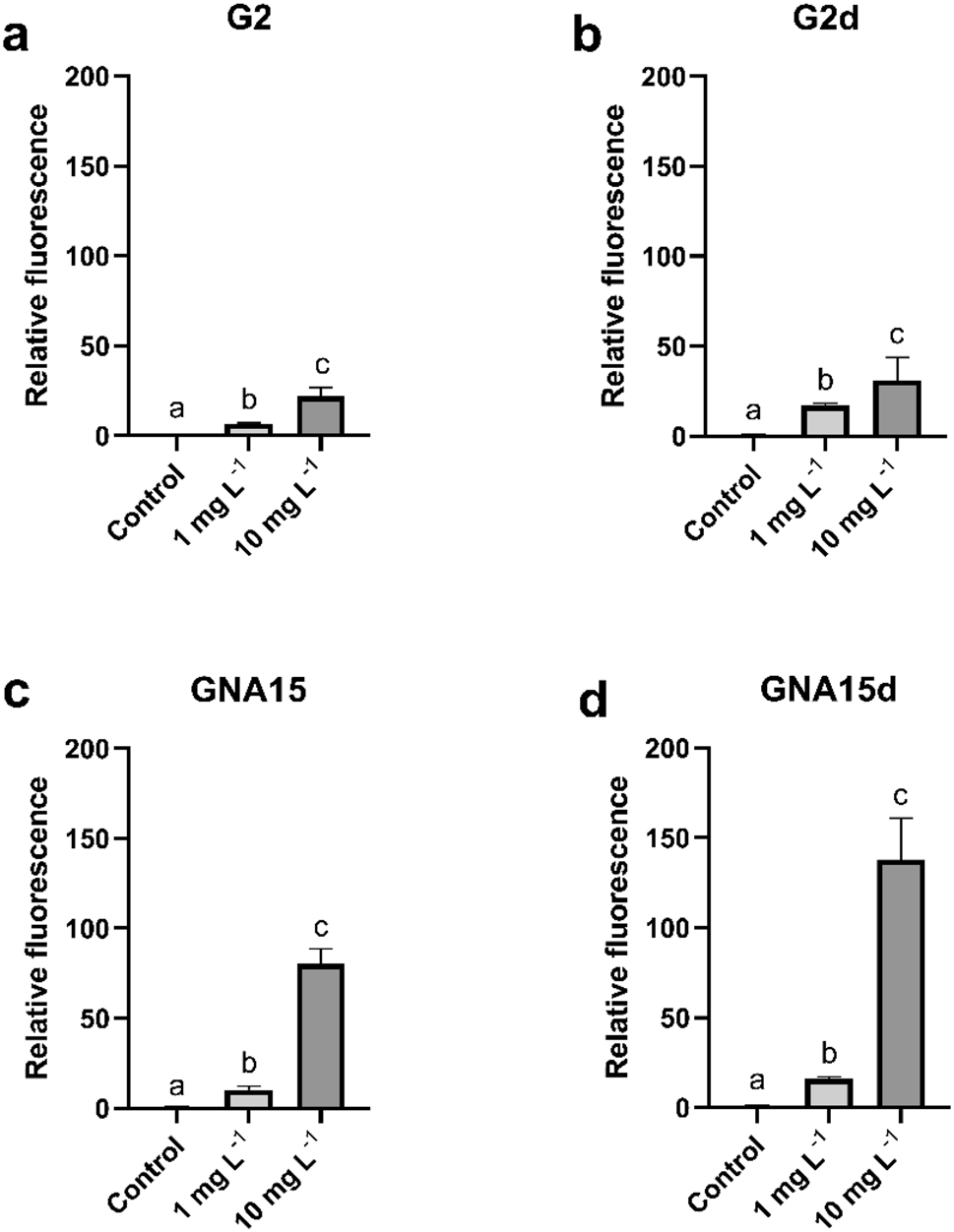
ROS of HT-29 cells exposed to different concentrations of G2 (**a**), G2d (**b**), G2d (**c**) and GNA15 (**d**) for 60 min. Results are expressed as % of control (untreated cells). Data represented the mean (± standard deviation, SD) of two independent experiments (where in total N = 6). Differences were established using a one-way ANOVA followed by multiple comparisons test (Tukey test) and considered significant when* p* ≤ 0.05. Different letters indicate significant differences between treatments.

As previously reported for A549 cells, after the maximum exposure time (t 60) (Fig. 7) GNA15 induced a higher increase in ROS levels than G2. Again, it was also observed that the degraded NMs induced the production of higher ROS levels in comparison with the pristine forms, particularly in the case of GNA15d. The comparison of the HT-29 ROS levels between different exposure conditions at t60, including their statistical significance, is displayed in Supplementary Tables S10 and S11.

### Determination of the irritation potential of pristine and degraded G2 and GNA15 using reconstructed human epidermis (RhE)

The In Vitro EpiDerm Skin Irritation Test was performed following the OECD 439 Test Guideline. In this case, we aimed to determine the irritant potential of all samples in stoichiometrically comparable graphene concentrations. Considering that GNA15 is composed of ~ 50% of G2, the tissues were exposed to 20 and 500 mg L^−1^ of G2 and G2d and 40 and 1000 mg L^−1^ of GNA15 and GNA15d (Supplementary Fig. S9). As shown in Supplementary Fig. S7, none of the NMs caused a reduction in the tissue viability over 50% when compared to the controls, and according to EU and Globally Harmonized System of Classification and Labelling Chemicals, GHS, (R38/ Category 2 or no label). The viability of the RhE was not significantly reduced in any of the exposure conditions when compared with the control condition. Therefore, none of the NMs could be considered irritants in the conditions tested.

The RhE media was collected after NMs exposure to evaluate the release of IL-1α by a specific ELISA assay. The IL-1α values obtained were 58.6 ± 34.0 pg mL^−1^ for non-exposed tissues, 194.4 ± 76.6 pg mL^−1^ for G2, 156.3 ± 147.9 pg mL^−1^ for G2d, 130.2 ± 29.8 pg mL^−1^ for GNA15 and 294.4 ± 89.1 pg mL^−1^ for GNA15d. Although an average increase in the production and release of IL-1α was observed in all the exposure conditions, being this increase more notable once exposed to GNA15d (294.4 pg mL^−1^), none of the observed differences were statically significant.

## Discussion

The use of engineered NMs has increased considerably during the last years, revealing the need to take action about potential occupational exposure issues related to these agents. The size, shape, and chemical and physical properties of NMs have a strong impact on human health. Also, their characteristics can change over time (e.g. due to degradation or transformation processes) and affect their toxicity. Humans can be exposed to NMs by inhalation, swallowing and skin/ocular deposition^28^, especially at manufacturing facilities. For this reason, it is imperative to investigate their potential effects on humans considering their fate and different exposure routes.^35–39^ In the environment, nanomaterials can suffer physical, chemical and biotransformation processes as a result of their exposure to different natural agents. The environmental factors can cause changes in the morphological structure, in the physical and chemical properties of the pristine NMs^40^, which may adversely affect to their toxicity. However, there are not many previous studies in the literature about the toxicity of transformed GFNs. Limited information is reported regarding the soil characteristics, particularly the soil chemical components and some parameters such as pH, soil type, cation exchange capacity, sunlight and microbes^41,42^. Nevertheless, there is no specific information available about the physical degradation of the GFNs. For that purpose, the present work is one of the first study where a physical degradation by ultrasonication has been reproduced, simulating the extreme environmental agents’ effect on these NPs after a long-term exposure period in the environment.

Upon ultrasound treatment of GNA15 and G2, we observed that the solutions of degraded NMs are more homogeneous than the solutions of pristine NMs. Comparing the physicochemical properties between pristine and degraded NMs, structural differences between G2 and GNA15 were appreciated, and additionally as expected, a change in shape and structural properties was observed when these NMs were subjected to degradation. TEM analysis showed that G2d and GNA15d appeared to display a higher degree of exfoliation than their original forms. In addition, smaller debris fragments were visible as well in degraded samples, especially in G2d (Fig. 2). On the other hand, significant morphological differences were observed between GNA15 and manganese oxide NPs, showing the last ones have round shape^43^. Raman analysis confirmed the structural changes after the ultrasonication, showing that the degraded NMs have a higher degree of exfoliation than their original forms (Fig. 3). The presence of exfoliated system could significantly affect the quality of the signal, this is reflected in the Raman spectra, where the degraded forms present a noisy signal. Regarding this, it was demonstrated that two dimensional (2D) materials-based, due to the surface plasmon resonance, enhance the Raman scattering. This effect could explain the enhanced signal collected for the ultrasonicated materials and further support the exfoliation. Moreover, we evaluated and compared their toxicological effects, employing different biological models representing the main exposure routes. We assessed previously the toxicity of Mn_3_O_4_ NPs on A549 and HT-29 cell lines, observing that these NPs affected A549 cell viability. However, they did not reduce significantly the HT-29 cell viability (see Table S12). As mentioned before, GNA15 contains in its composition MnOx-NPs. Considering this, it would be expected that GNA15 NM resulted in more toxicity than G2. However, the obtained results suggested that the bare graphene provoked a higher mortality rate than its MnOx-NPs decorated version (see Table S12). Another interesting finding is that the degraded forms are more hazardous, as they resulted to have a higher toxicological impact^44^ than their pristine counterparts in both viability and ROS determination assays. This could also be attributed to the fact that the ultrasonication process promotes the NMs exfoliation and the release of metal ions, which are related to the toxicological effects on the cells.

Several factors such as the dispersion, the susceptibility to release ions or the ability of NPs to be uptaken and internalised by the cells can influence their toxicological potential. After the physical degradation, the size of the particles decreases, which could facilitate their cellular uptake, and therefore, increase the risk for human health^19^. The toxicological potential of the NMs is related to their capacity to enter into the cells. The structure and the physicochemical properties of the NMs, as well as the cell type tested or the cellular differentiation status, play an important role in cellular uptake^45^.

According to the literature, graphene NPs can enter into lung epithelial cells. Li et al. explained this process proposing as a mechanism the local piercing caused by the sharp protrusions that are abundant in the irregular edges of the graphene, a process by which even large multilayer sheets of micrometre-scale lateral dimension could enter the cells. By the same token^46^, Jin et al.^47^ demonstrated that graphene oxide can enter into A549 cells, being located in the cytoplasm and nucleus, without affecting the viability of the cells. Furthermore, Šestáková et al.^48^ determined by TEM the presence of graphene platelets in A549 cells after an 8-week exposure, suggesting that the cells can enclose graphene platelets, which were free in the cytoplasm into the membrane-bound vesicles. Regarding HT-29 cells, recently it was described the internalisation of graphene oxide (GO) by differentiated cells^49^. All this information suggests that the NPs used in this work are internalised by the cells, leading to the cell damage observed. DLS analysis did not show a clear reduction in particle size between pristine and degraded forms, which is not surprising, as this technique could overestimate the particle sizes of the sample^50^. However, opposite to what was observed for GNA15, GNA15d shows a smaller average size in the culture medium than in water, indicating the formation of less aggregates and higher stability in the degraded form. PDI values of both NMs in culture medium (lower in case of GNA15d), indicate a narrower particle size distribution for GNA15d, suggesting less aggregation as well. In addition, the results are in line with Raman and TEM analyses. Where the degraded materials showed a higher degree of exfoliation in comparison with their original forms. This fact could facilitate the internalisation of the materials by the cells. These observations support the viability results, which indicated that the degraded forms reduce more the cell viability than the pristine materials. On the other hand, the reduction of the cell viability could be related with the presence of the metal ions in the medium. In this regard, we determined by ICP-MS if the degradation process facilitated the release of metal ions. The ICP-MS analysis showed that GNA15 released more Mn ions than G2 (as expected). However, it was observed that the availability of Mn ions in the culture medium does not increase the toxic effects of GNA15. That is especially relevant in biological systems because ions could be released into the cytoplasm or the interstitial fluid, being far more mobile than the nanoparticles themselves. If cells become saturated with concentrations of metals over the homeostatic levels, a range of detrimental effects may be triggered^51^. Moreover, testing degraded materials has also important considerations for waste management, where potential health security policies may be needed to protect workers. We realised that the induced ROS response is significantly higher when cells are exposed to GNA15 when compared to G2, as mentioned earlier, and this is probably related to the presence of MnOx-NPs in GNA15, due to the strong oxidative stress-inducing properties of this metal oxide^43^. High oxidative stress is contributing to the cytotoxicity provoked by NPs, especially for metal oxide particles such as TiO_2_, ZnO, Co_3_O_4_, and CuO^52,53^. ROS can damage DNA, lipids and protein, deplete the antioxidant status, or induce apoptosis^52,53^. We found no previous toxicology studies (including those reporting ROS accumulation) focusing on graphene decorated with MnOx-NPs in any mammal cellular model, and only a handful related to the toxicity of MnO_2_ NPs. Hafez et al. have demonstrated that MnO_2_ NPs significantly increase the level of ROS through mitochondria I and III complexes^54^. Fernández-Pampín et al. showed that Mn_3_O_4_ NPs provoke the ROS generation in A549 and HT-29 cell lines.^43^ Moreover, a recent study by Stygar et al.^55^ determined the toxicity of GO in comparison with an Mn^2+^ contaminated GO over human dermal fibroblasts and A549 cells. By measuring selected oxidative stress markers, the authors reported that GO induced a lower oxidative stress response in comparison with the contaminated NM, but none of the materials caused the cellular collapse. With further caution, due to the evident differences between both studies, the observations indirectly support each other results.

As stated previously, despite the higher ROS response exerted by GNA15 when compared to G2, we observed an opposite trend regarding their impact on the viability of A549 and HT29 cells. It must be noticed that while the viability assay is performed after 24 h of cell exposure, the ROS determination assay is performed after 1 h of cell exposure, following standard procedures. Considering this, the obtained results indicate that both cell models could overcome the increase in ROS levels, limiting the relevance of this mechanism in the viability reduction caused by G2 and GNA15.

In contrast to what was observed in the cell lines related to inhalation and ingestion, we did not observe any viability loss on the 3D RhE model in the presence of G2 or GNA15, even if the maximum concentration employed from both NMs in the exposure experiment was remarkably higher. However, the RhE model consists of a fully differentiated epidermis including the *stratum basale*, *stratum spinosum*, *stratum granulosum* and *stratum corneum*. This model resembles the human skin tissues with layers and boundaries that protect the inner layers of the epidermis^56^, while the used lung and gastrointestinal models consist of cell monolayers, which seem to be more susceptible to suffer damage upon direct contact with NMs. We also showed that RhE exposed to the pristine and degraded NMs did not release significantly higher IL-1α, verifying that these NMs are not skin irritants. According to European Union Reference Laboratory for alternatives to animal testing (EURL ECVAM)^57^, although the accuracy of irritant classification is not improved by measuring IL-1α release, its determination may be useful to better classify the mild to moderate irritancy potential of a test substance. Our results agree with Fusco et al.^56^, who demonstrated that graphene material is not irritant by itself. These authors describe that in some cases the graphene materials can produce skin irritability due to the presence of surfactant residues in the final product, which are employed to facilitate the dispersion of the graphene.

Our study is relevant to have a better understanding of the potential hazard of novel multicomponent nanomaterials, providing insights as well on their toxicity along their life cycle (considering their potential degradation) and about mixture toxicity effects, which are related to the compositions and integrity of the NMs, and also with the route of exposure. The main risks associated with human exposure to the studied NMs are related to the occupational perspective, where there is a higher chance of inhalation, swallowing and skin/ocular deposition. Notwithstanding, the design of novel applications using graphene or functional graphene, such as biomimetic epidermal sensors^58^ for electrophysiological monitoring^59^, will facilitate the direct contact of the NMs with the skin. In the present study, we have confirmed that graphene is not irritant for the skin, demonstrating for the first time that its functionalization with MnOx-NPs yields an equaly safe product. Specifically, all tested materials in the studied conditions are not irritants according to OECD TG 439 guideline.

## Conclusions

The present research work provides novel insights into the physicochemical and toxicological properties of a novel multicomponent nanomaterial (GNA15) composed of graphene and manganese oxide NPs, considering potential risks associated with human exposure through different routes, considering how the addition of MnOx-NPs influence the potential toxicity of graphene, and also its potential degradation along its life cycle. We have compared GNA15 susceptibility to degradation/metal ions release after a heavy ultrasonication treatment with that of the graphene product employed for its production (G2), as well as the properties of the pristine and degraded forms. The comparative toxicological assessment between pristine and degraded GNA15 and G2 suggests that both the composition and integrity of the NMs are relevant factors in their toxicity, which can vary as well depending on the exposure route. While the viability of respiratory and gastrointestinal models was reduced in a dose-dependent manner in the presence of both GNA15, GNA15d, G2 and G2d, none of the materials produced irritant effects on 3D reconstructed skin in the studied conditions. Also, GNA15d and G2d induced a higher cytotoxicity effect in the lung and gastrointestinal models. Finally, in terms of cell viability, the addition of MnOx-NPs does not represent a negative impact. Therefore, we can conclude that the novel multicomponent nanomaterial with enhanced energy storage properties is less cytotoxic than graphene, even after the degradation process. Moreover, the obtained results are very valuable to better understand the potentially harmful effects of complex NMs such as graphene decorated with metal oxide NPs, along their life cycle.

## Material and methods

### Pristine and degraded NM forms

Materials were provided by Gnanomat (Madrid, Spain): pristine graphene nanoplatelets (G2), and graphene functionalized with MnO_x_-NPs (GNA15). The latter NM is composed of 47% of G2 and 53% of MnO_x_-NPs, synthesised by the deposition of MnO_x_-NPs in G2 through a nanotechnological patented process (Patent number ES2678419A1).

To obtain the degraded forms (G2d and GNA15d), NMs resuspended in ultrapure water (4 mg mL^−1^) were subjected to sonication for 3 h, using pulsed mode (pulse duration: 5 s, rest: 2 s), with a tip sonicator (Sonics, 20 kHz; 500 W; amplitude: 30%), using a titanium alloy solid tip (diameter: 13 mm).

Stocks dispersions were prepared in distilled H_2_O (_d_H_2_O) at 4 mg mL^−1^ and they were sonicated in a water bath for 20 min before being stored at 4 °C until experiments. Prior to the exposure assessment, the stocks suspensions were vortex in order to facilitate their dissolution. Then, serial dilutions were prepared from homogenous stock solution.

The tested concentrations (1, 5 and 10 mg L^−1^) were selected because at higher concentrations it was observed a few aggregates in the cell culture media.

### NM physicochemical characterisation

GNA15 was characterised by thermogravimetric analysis (TGA) using a TGA analyser (METTLER TOLEDO TGA-2 Start system) under environmental conditions. The operating temperature ranged from 40 to 1050 °C with an air flow rate of 100 mL min^−1^ and a heating-rate of 10 °C min^−1^.

The chemical composition and crystalline phase of composite materials were determined by X-ray diffraction analysis, using a Rigaku SmartLab X-ray Diffractometer with a Bragg–Brentano geometry, Cu Kα radiation = 1.54178 Å, equipped with a graphite monochromator in the diffracted beam. Powders were dispersed, and then compacted into an amorphous glass support used as sample holder for measuring. Semiquantitative evaluation of phase abundance and structural features were estimated by nonlinear least-square refinement procedure, according to Rietveld method, using the MAUD (Materials Analysis Using Diffraction) software^60^. For G2 pattern the asymmetric peak around 26°, corresponding to (002) reflections was analysed by using two theoretical gaussian peaks. The crystallite size, *D*_*002*_*,* was then determined by the Debye–Scherrer equation and the number of graphene layers, *n*, was obtained by the following formula:

$$n=\frac{{D}_{002}}{{d}_{002}}$$

where *d*_*002*_ is the interlayer distance calculated according to the Bragg equation.

DLS and the ζ-potential determination were done using a Zetasizer ZS90 (Malvern Panalytical, UK). Before performing the analyses, an aliquot of the sample is sonicated for 5 min, and then it is diluted 1: 4 in water (50 mg mL^−1^). TEM analyses were performed at the Microscopy Unit at the University of Valladolid. Samples were deposited on Lacey Carbon Type-A, 300 mesh, copper grids, and visualised and photographed using a JEOL JEM-1011 HR TEM coupled with a Gatan Erlangshen ES1000W camera.

Raman measurements were performed in a “Senterra” Raman microscope (Bruker) under a laser excitation of 532 nm (12.5 mW power). The spectra were collected with a resolution of ~ 3–5 cm^−1^ and an integration time of 30 s. The samples were deposited on a silicon wafer by drop casting.

We prepared 4 mg mL^−1^ solutions of all NMs into _d_H_2_O for the ion release measurement, where colloidal suspensions were the starting point for degraded materials. Solutions were centrifuged for 20 min at 5000 rpm. Three mL from supernatants were collected and analysed as well as pristine solid G2 and GNA15. We used an Agilent 8900 ICP-QQQ instrument, at normal hot plasma conditions. In addition, the same protocol was employed to determine the ions concentration in the supernatants of the different NMs solutions. In this case, the solutions were prepared in culture media with 1% FBS at a higher concentration used in the in vitro viability assays, 10 mg L^−1^. Extraction lens voltages were optimised for maximum sensitivity using an Agilent 1 ppb tuning solution containing Li, Y, Ce and Tl. The operating parameters were: RF power 1550 W, 10.0 mm of sampling depth, nebuliser gas flow rate 1.07 L min^−1^, auxiliary gas flow rate 0.90 L min^−1^, extraction lens 1 − 10.0 V, while the extraction lens 2 − 250 V, Omega lens bias − 150 V, Omega lens 10.2 V. Samples were digested using 0.02 g and 7 mL of HNO_3_ and 1 mL of HCl (30%), in a closed vessel within an ETHOS SEL (Milestone, Italy) microwave digestor.

### Cell lines

We used A549 (ATCC, CCL-185) as a model for lung tissue, HT-29 (ATCC, HTB-38) as an intestinal model, and a three-dimensional reconstructed epidermal tissue, normal human-derived epidermal keratinocytes (NHEK) (MatTek Life Sciences, USA). A549 cells were grown in DMEM supplemented with 10% (v/v) fetal bovine serum (FBS) (Invitrogen, USA), and 1% penicillin and streptomycin. Growth media for HT29 cells was McCoy’s 5A, 10% (v/v) FBS (Invitrogen, USA), 1% penicillin–streptomycin and 1% l-glutamine. NHEK was grown according to the manufacturer’s instructions. Incubation at 37 °C, 5% CO_2_ humidified environment.

### Viability assay: MTT

Cell viability was tested by 3-(4,5-dimethyl-2-thiazolyl)-2,5-diphenyl-2H-tetrazolium bromide (MTT) assay, based on the reduction of MTT to an insoluble purple-coloured formazan salt by mitochondrial enzymes of viable cells^61,62^. Plates were seeded with 5 × 10^3^ cells per well (A549) or 1 × 10^4^ cells per well (HT-29), and left for incubation at 37 °C for 24 h at 5% CO_2_ environment. After incubation, media was removed and wells were washed once with sterile PBS. Assayed concentrations were added to the corresponding wells, where untreated wells received cultivation media (negative control), and positive control wells received sterile H_2_O (positive control was not showed in the figures). All solutions were prepared in the corresponding growth media, but 1% FBS (v/v) to prevent the FBS effect, with no antibiotic modifications. Incubation was carried out for 24 h at a continuous 37 °C, 5% CO_2_ environment. After 24 h exposure, media with NMs was retrieved. A hundred µL of growth media supplemented with 0.5 mg mL^−1^ MTT were added per well, and removed after 3 h incubation. Formed formazan crystals during incubation were solubilised with 100 µL DMSO, and incubated for 15 min at 37 °C in darkness, continuous shaking. We measured absorbance at 590 nm with a microplate reader (BioTek Synergy HT). Three biological replicates were included in each independent assay.

### Oxidative stress assay

The method^23^ was adapted for human cell lines by Fernández-Pampín et al.^30,43^, and quantitatively measures ROS in cells exposed to NMs. We seeded A549 and HT-29 cells in 96 micro-well plates, at a density of 3 × 10^4^ cells per well. Cells were incubated for 24 h and washed once with 1 × Hank’s Balanced Salt Solution (HBSS) pH 7.2 (NaOH adjusted). Then, the cells were incubated with 50 µM 2,7-dichlorofluorescein diacetate (DCFH-DA) for 30 min at 37 °C in darkness, except for three wells incubated with HBSS without dye, which were employed as blanks. Upon incubation, we washed all wells once with HBSS. Two-hundred µL of previously prepared NMs dissolved in HBSS were added to each well at different concentrations ranging from 1 to 10 mg L^−1^, being the positive control cells exposed to 20 µM H_2_O_2_ and the negative control cells incubated with HBSS alone. We measured fluorescence in a microplate reader (BioTek Synergy HT, excitation wavelength 485/20 and emission wavelength 520/20). Fluorescence was recorded at 0, 30, and 60 min. Three biological replicates were included in each independent assay.

### In Vitro EpiDerm Skin Irritation Test (EPI-200-SIT)

For skin model exposure assays, we used In Vitro EpiDerm Skin Irritation Test, EPI-200-SI (MatTek). Firstly, the inability of the NMs to interfere with and/or to reduce the MTT was confirmed following the guideline suggestions.

Then, the test was performed according to the manufacturer’s recommendations. This test fulfils the criteria of OECD TG439. Tissues exposed to DPBS were used as a negative control and as a positive control it was employed tissues exposed to 5% SDS. Positive control was not showed in the figures.

### IL-1α quantification

Culture media from NHEK was collected and stored at − 80 °C. The cytokine concentration was evaluated by ELISA test (Diaclone, France) following the manufacturer’s instructions.

### Statistical analysis

The results are represented as mean ± SD. Differences between the different treatments were established using a one-way ANOVA followed by a multiple comparisons test (Tukey test). Statistical test was carried out using GraphPad Prism Software, Inc (version 8.0.2). Statistical significance was considered at *p* ≤ 0.05.

## Supplementary Information

Supplementary Information.

## Data Availability

The datasets generated and analysed during the current study are available from the corresponding author upon reasonable request.
