# Supplementary material for: Toxicological assessment of pristine and degraded forms of graphene functionalized with MnOx nanoparticles using human in vitro models representing different exposure routes
Source: Sci Rep. 2023 Jul 22;13:11846. doi: 10.1038/s41598-023-38993-y (PMC10363126; doi:10.1038/s41598-023-38993-y)
Supplement: Supplementary file 1 — Supplementary Information. [file 41598_2023_38993_MOESM1_ESM.docx]

**Supplementary file**

**Toxicological assessment of pristine and degraded forms of MnOx-functionalized graphene using human *in vitro* models representing different exposure routes**

Natalia Fernández-Pampín^1*^, Juan José González Plaza^1*^, Alejandra García-Gómez^2b^, Elisa Peña^2b^, Sebastiano Garroni^3^, Matteo Poddighe^4^, Carlos Rumbo^1^, Rocío Barros^1^, Sonia Martel^1^, Santiago Aparicio^1^, Juan Antonio Tamayo Ramos^1*^

^1^ International Research Centre in Critical Raw Materials-ICCRAM, University of Burgos, Plaza Misael Bañuelos s/n, 09001 Burgos, Spain

^2^ Gnanomat, C/Faraday 7, 28049 Madrid, Spain

^3^ Department of Chemical, Physics, Mathematics and Natural Science, University of Sassari, Via Vienna 2, 07100 Sassari, Italy

^4^ Laboratory of Materials Science and Nanotechnology (LMNT), Department of Chemical, Physics, Mathematics and Natural Science, University of Sassari, CR-INSTM, Via Vienna, 2, 07100 Sassari, Italy

* Equal contribution

† Corresponding author:

Juan Antonio Tamayo-Ramos:

Mailing address: International Research Center in Critical Raw Materials-ICCRAM, Universidad de Burgos, Plaza Misael Bañuelos s/n, 09001, Burgos, Spain.

Email: [jatramos@ubu.es](mailto:jatramos@ubu.es)

**Table S1.** Structural and Microstructural parameters determined by Rietveld refinement applied to the pattern reported in Figure S1. Wt.: Weight.

| **Phase** | **a (Å)** | **b (Å)** | **c (Å)** | **Crystallite size (Å)** | **Microstrain** | **Wt.%** |
| --- | --- | --- | --- | --- | --- | --- |
| **Mn_3_O_4_** | 5.7659 | -- | 9.4713 | 1022 | 6.78 E-6 | 67.8 |
| **Mn_2_O_3_** | 9.4196 | -- | -- | 976 | 9.14 E-4 | 32.2 |

**Table S2.** Structural and Microstructural parameters determined by Rietveld refinement applied to the pattern reported in Figure 1c.

| **Phase** | **a (Å)** | **b (Å)** | **c (Å)** | **Crystallite size (Å)** | **Microstrain** |
| --- | --- | --- | --- | --- | --- |
| **Graphene** | 2.5624 | -- | 6.7750 | 500 | 1.89 E-2 |
| **Mn_2.03_O_4_ (cubic)** | 8.0666 | -- | -- | 154 | 3.37 E-3 |
| **Mn_2_O_5.44_ (hexagonal)** | 2.8375 |  | 14.3805 | 70 | 1.78 E-2 |

**Table S3.** ICP-MS analysis of elements in NMs suspensions (4 mgmL^-1^) supernatants. Quantities are expressed in ppb (parts per billion).

|  | **G2**  **supernatant** | **G2d**  **supernatant** | **GNA15**  **supernatant** | **GNA15d**  **supernatant** |
| --- | --- | --- | --- | --- |
| **Mn** | 0.05 | 0.24 | 99.68 | 5,690.27 |
| **Fe** | 0.52 | 1.18 | 0.93 | 38.7 |
| **Ni** | 0.06 | 0.06 | 0.06 | 2.2 |
| **Cu** | 0.13 | 2.98 | 0.23 | 1.02 |
| **Zr** | 0.01 | 0.21 | 0.36 | 10.44 |

**Table S4.** Ratios between the A549 viability values recorded upon 24 h exposure to the tested materials. Ratios were calculated by dividing the percentage of survival for material A, by the percentage of survival of material B. A ratio A/B over 1 indicates that cells showed higher survival rates in material A in comparison with material B. Inverse ratios have been calculated for comparison purposes, in case that ratios were lower than 1.

|  | **Ratio** | **Inverse ratio** |
| --- | --- | --- |
| 1 mg L^-1^ |  |  |
| **G2/GNA15** | 0.90 | 1.10 |
| **G2d/GNA15d** | 0.77 | 1.29 |
| **G2/G2d** | 1.34 | 0.74 |
| **GNA15/GNA15d** | 1.14 | 0.87 |
|  |  |  |
| 5 mg L^-1^ |  |  |
| **G2/GNA15** | 0.74 | 1.34 |
| **G2d/GNA15d** | 0.86 | 1.15 |
| **G2/G2d** | 1.19 | 0.83 |
| **GNA15/GNA15d** | 1.39 | 0.7 |
|  |  |  |
| 10 mg L^-1^ |  |  |
| **G2/GNA15** | 0.84 | 1.18 |
| **G2d/GNA15d** | 0.90 | 1.11 |
| **G2/G2d** | 1.16 | 0.85 |
| **GNA15/GNA15d** | 1.46 | 0.68 |

**Table S5.** Comparison between the A549 cytotoxic values obtained in the MTT assay, upon 24 h exposure to the tested materials. The assayed concentrations are indicated in italics at the uppermost left corner. The adjusted *p*-value is shown. Absence of statistical differences between the response produced by different materials is indicated (ns).

| *1 mg L^-1^* | **G2** | **G2d** | **GNA15** | **GNA15d** |
| --- | --- | --- | --- | --- |
| **G2** |  | ns | ns | ns |
| **G2d** |  |  | ns | ns |
| **GNA15** |  |  |  | ns |
| **GNA15d** |  |  |  |  |
|  |  |  |  |  |
| *5 mg L^-1^* | **G2** | **G2d** | **GNA15** | **GNA15d** |
| **G2** |  | ns | ˂0.05 | ns |
| **G2d** |  |  | <0.05 | ns |
| **GNA15** |  |  |  | <0.05 |
| **GNA15d** |  |  |  |  |
|  |  |  |  |  |
| *10 mg L^-1^* | **G2** | **G2d** | **GNA15** | **GNA15d** |
| **G2** |  | ns | ˂0.05 | ˂0.05 |
| **G2d** |  |  | <0.0001 | ns |
| **GNA15** |  |  |  | <0.0001 |
| **GNA15d** |  |  |  |  |

**Table S6.** Ratios between the A549 ROS response induced upon exposure to the tested materials at t60. Ratios were calculated by dividing the fluorescence for material A, by fluorescence of material B. A ratio A/B over 1 indicates that cells showed higher ROS rates with material A in comparison with material B. Inverse ratios have been calculated for comparison purposes, in case that ratios were lower than 1.

|  | **Ratio** | **Inverse ratio** |
| --- | --- | --- |
| 1 mg L^-1^ |  |  |
| **G2/GNA15** | 0.53 | 1.89 |
| **G2d/GNA15d** | 0.90 | 1.11 |
| **G2/G2d** | 0.51 | 1.96 |
| **GNA15/GNA15d** | 0.87 | 1.15 |
|  |  |  |
| 10 mg L^-1^ |  |  |
| **G2/GNA15** | 0.28 | 3.57 |
| **G2d/GNA15d** | 0.38 | 2.66 |
| **G2/G2d** | 0.57 | 1.77 |
| **GNA15/GNA15d** | 0.76 | 1.32 |

**Table S7.** Comparison between the A549 ROS response induced upon exposure to the tested materials at t60. The assayed concentrations are indicated in italics at the uppermost left corner. The adjusted *p* -value is shown. Absence of statistical differences between the response produced by different materials is indicated (ns).

| 1 mg L^-1^ | **G2** | **G2d** | **GNA15** | **GNA15d** |
| --- | --- | --- | --- | --- |
| **G2** |  | <0.0001 | <0.0001 | <0.0001 |
| **G2d** |  |  | ns | 0.039 |
| **GNA15** |  |  |  | 0.0059 |
| **GNA15d** |  |  |  |  |
|  |  |  |  |  |
| 10 mg L^-1^ | **G2** | **G2d** | **GNA15** | **GNA15d** |
| **G2** |  | 0.0009 | <0.0001 | <0.0001 |
| **G2d** |  |  | <0.0001 | <0.0001 |
| **GNA15** |  |  |  | <0.0001 |
| **GNA15d** |  |  |  |  |

**Table S8.** Ratios between the HT29 viability values recorded 24 h, upon exposure to the tested materials. Ratios were calculated by dividing the percentage of survival for material A, by the percentage of survival of material B. A ratio A/B over 1 indicates that cells showed higher survival rates in material A in comparison with material B. Inverse ratios have been calculated for comparison purposes, in case that ratios were lower than 1.

|  | **Ratio** | **Inverse ratio** |
| --- | --- | --- |
| 1 mg L^-1^ |  |  |
| **G2/GNA15** | 0.88 | 1.12 |
| **G2d/GNA15d** | 0.82 | 1.20 |
| **G2/G2d** | 1.19 | 0.84 |
| **GNA15/GNA15d** | 1.11 | 0.90 |
|  |  |  |
| 5 mg L^-1^ |  |  |
| **G2/GNA15** | 0.73 | 1.36 |
| **G2d/GNA15d** | 1.7 | 0.58 |
| **G2/G2d** | 0.64 | 1.54 |
| **GNA15/GNA15d** | 1.23 | 0.8 |
|  |  |  |
| 10 mg L^-1^ |  |  |
| **G2/GNA15** | 0.59 | 1.67 |
| **G2d/GNA15d** | 0.75 | 1.32 |
| **G2/G2d** | 1.22 | 0.81 |
| **GNA15/GNA15d** | 1.55 | 0.64 |

**Table S9.** Comparison between the HT29 cytotoxic values obtained in the MTT assay, upon 24 h exposure to the tested materials. The assayed concentrations are indicated in italics at the uppermost left corner. The adjusted *p* -value is shown. Absence of statistical differences between the response produced by different materials is indicated (ns).

| 1 mg L^-1^ | **G2** | **G2d** | **GNA15** | **GNA15d** |
| --- | --- | --- | --- | --- |
| **G2** |  | ns | ns | ns |
| **G2d** |  |  | <0.05 | ns |
| **GNA15** |  |  |  | ns |
| **GNA15d** |  |  |  |  |
|  |  |  |  |  |
| 5 mg L^-1^ | **G2** | **G2d** | **GNA15** | **GNA15d** |
| **G2** |  | ˂0.05 | ˂0.05 | ns |
| **G2d** |  |  | <0.0001 | <0.0001 |
| **GNA15** |  |  |  | <0.05 |
| **GNA15d** |  |  |  |  |
|  |  |  |  |  |
| 10 mg L^-1^ | **G2** | **G2d** | **GNA15** | **GNA15d** |
| **G2** |  | ˂0.05 | <0.0001 | ns |
| **G2d** |  |  | <0.0001 | <0.05 |
| **GNA15** |  |  |  | <0.0001 |
| **GNA15d** |  |  |  |  |

**Table S10.** Ratios between the HT29 ROS response induced upon exposure to the tested materials at t60. Ratios were calculated by dividing the fluorescence for material A, by fluorescence of material B. A ratio A/B over 1 indicates that cells showed higher ROS rates with material A in comparison with material B. Inverse ratios have been calculated for comparison purposes, in case that ratios were lower than 1.

|  | **Ratio** | **Inverse ratio** |
| --- | --- | --- |
| 1 mg L^-1^ |  |  |
| **G2/GNA15** | 0.65 | 1.53 |
| **G2d/GNA15d** | 1.05 | 0.95 |
| **G2/G2d** | 0.40 | 2.50 |
| **GNA15/GNA15d** | 0.64 | 1.56 |
|  |  |  |
| 10 mg L^-1^ |  |  |
| **G2/GNA15** | 0.27 | 3.64 |
| **G2d/GNA15d** | 0.23 | 4.43 |
| **G2/G2d** | 0.71 | 1.41 |
| **GNA15/GNA15d** | 0.58 | 1.72 |

**Table S11.** Comparison between the HT29 ROS response induced upon exposure to the tested materials at t60. The assayed concentrations are indicated in italics at the uppermost left corner. The adjusted *p*-value is shown. Absence of statistical differences between the response produced by different materials is indicated (ns).

| 1 mg L^-1^ | **G2** | **G2d** | **GNA15** | **GNA15d** |
| --- | --- | --- | --- | --- |
| **G2** |  | <0.0001 | 0.0007 | <0.0001 |
| **G2d** |  |  | <0.0001 | ns |
| **GNA15** |  |  |  | <0.0001 |
| **GNA15d** |  |  |  |  |
|  |  |  |  |  |
| 10 mg L^-1^ | **G2** | **G2d** | **GNA15** | **GNA15d** |
| **G2** |  | ns | <0.0001 | <0.0001 |
| **G2d** |  |  | <0.0001 | <0.0001 |
| **GNA15** |  |  |  | <0.0001 |
| **GNA15d** |  |  |  |  |

**Table S12.** Viability of A549 and HT29 cells exposed to different concentrations of the tested materials and Mn_3_O_4_ NPs for 24 h. Results are expressed as % of control (untreated cells). Data represented the mean and the standard deviation (SD) of two independent experiments.

|  | **A549** | **HT29** | **A549** | **HT29** | **A549** | **HT29** |
| --- | --- | --- | --- | --- | --- | --- |
|  | **1 mg L^-1^** | | **5 mg L^-1^** | | **10 mg L^-1^** | |
| **Mn_3_O_4_** | 91.8 ± 9.2 | 96.9 ± 10.2 | 82.9 ± 14.3 | 96.8 ± 7.9 | 77.6 ± 6.7 | 97.0 ± 5.9 |
| **G2** | 73.5 ± 10.6 | 85.9 ± 15.5 | 49.0 ± 10.1 | 62.1 ± 4.9 | 43.5 ± 5.8 | 42.5 ± 3.3 |
| **G2d** | 54.6 ± 10.6 | 72.1 ± 6.6 | 41 ± 5.3 | 40.4 ± 3.0 | 37.3 ± 8.1 | 34.6 ± 7.6 |
| **GNA15** | 81.1 ± 10.5 | 96.8 ± 11.2 | 66.1 ± 10.9 | 85.1 ± 11.0 | 51.4 ± 4.8 | 71.4 ± 6.6 |
| **GNA15d** | 70.6 ± 9.1 | 87.1 ± 10.9 | 47.4 ± 7.4 | 68.8 ± 7.2 | 35.2 ± 6.8 | 46.0 ± 8.0 |

*The control values are represented in the figures are included in the text.

**Table S13.** ROS of A549 and HT29 cells exposed to different concentrations of different concentrations of the tested materials and Mn_3_O_4_ NPs for 60 minutes. Results are expressed as % of control (untreated cells). Data represented the mean and the standard deviation (SD) of two independent experiments.

|  | **A549** | **HT29** | **A549** | **HT29** |
| --- | --- | --- | --- | --- |
|  | **1 mg L^-1^** | | **10 mg L^-1^** | |
| **Mn_3_O_4_** | 1.9 ± 0.2 | 2.5 ± 0.1 | 10.4 ± 2.0 | 16.7 ± 1.0 |
| **G2** | 4.1 ± 0.3 | 6.7 ± 0.6 | 17.3 ± 2.3 | 22.0 ± 4.9 |
| **G2d** | 8.1 ± 0.5 | 16.9 ± 1.4 | 31.2 ± 3.4 | 31.1 ± 12.7 |
| **GNA15** | 7.8 ± 0.6 | 10.3 ± 1.9 | 63.1 ± 11.4 | 80.2 ± 8.4 |
| **GNA15d** | 9.0 ± 0.6 | 16.1 ± 1.0 | 83.1 ± 10.1 | 137.8 ± 23.0 |

*The control values are represented in the figures are included in the text.

**Table S14.** Viability of EpiDerm tissues exposed to different concentrations of the tested materials and Mn_3_O_4_ NPs for 1 h. Results are expressed as % of control (untreated cells). Data represented the mean and the standard deviation (SD) of two independent experiments.

|  | **20 mg L^-1^** | **40 mg L^-1^** | **500 mg L^-1^** | **1000 mg L^-1^** |
| --- | --- | --- | --- | --- |
| **Mn_3_O_4_** |  | - | 100.0 ± 3.7 | - |
| **G2** | 94.6 ± 10.7 | - | 91.3 ± 14.1 | - |
| **G2d** | 98.3 ± 3.1 | - | 99.6 ± 2.0 | - |
| **GNA15** | - | 100.5 ± 25.3 | - | 91.7 ± 2.5 |
| **GNA15d** | - | 116.02 ± 13.5 | - | 90.9 ± 3.7 |

*The control values are represented in the figures are included in the text.


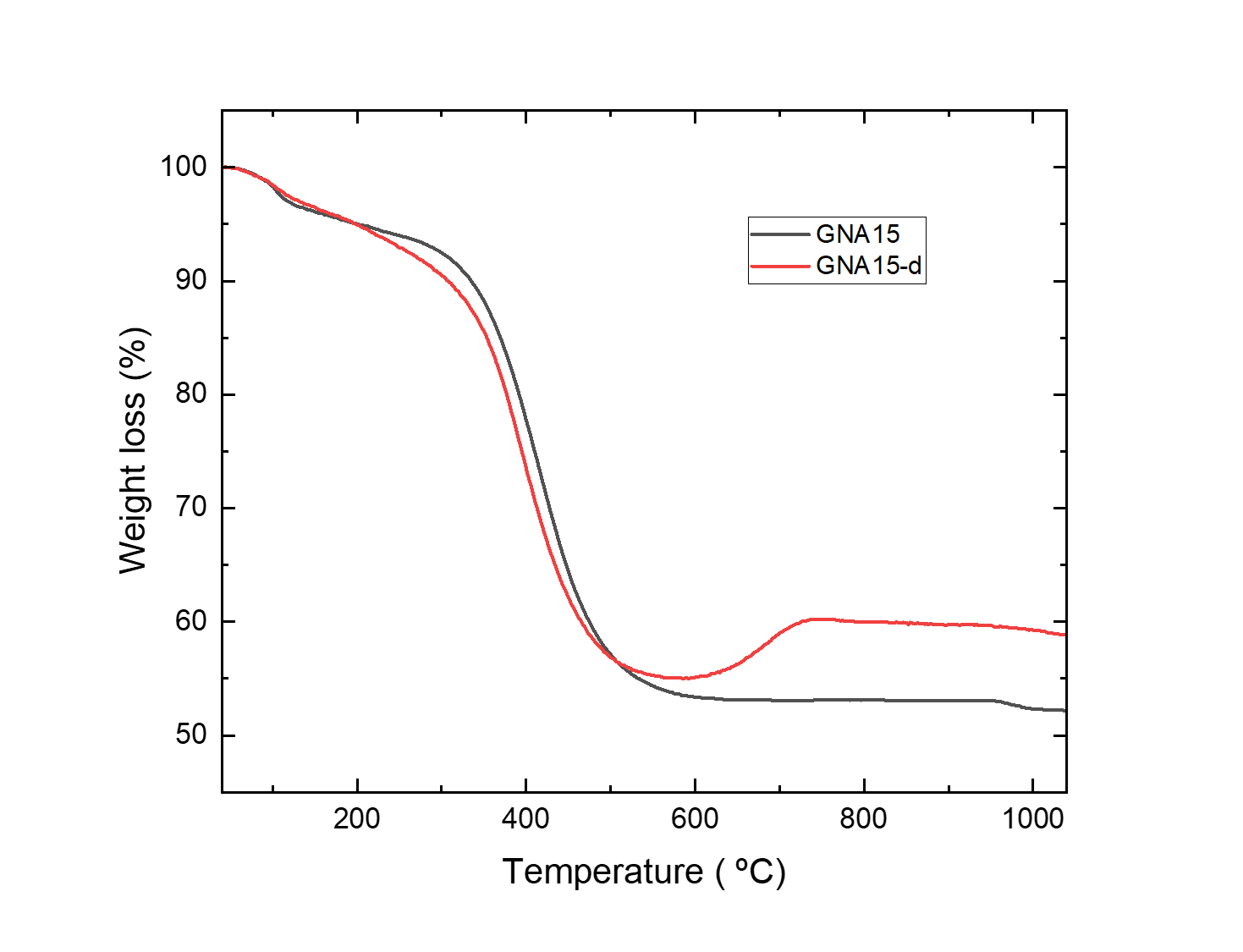


**Figure S1.** TGA analysis of GNA15 and GNA15d in air conditions. The composition of the nanomaterials was analysed with the mass reduction as a function of temperature.





**Figure S2.** XRD pattern of the MnOx materials used for the synthesis of GNA15.

**
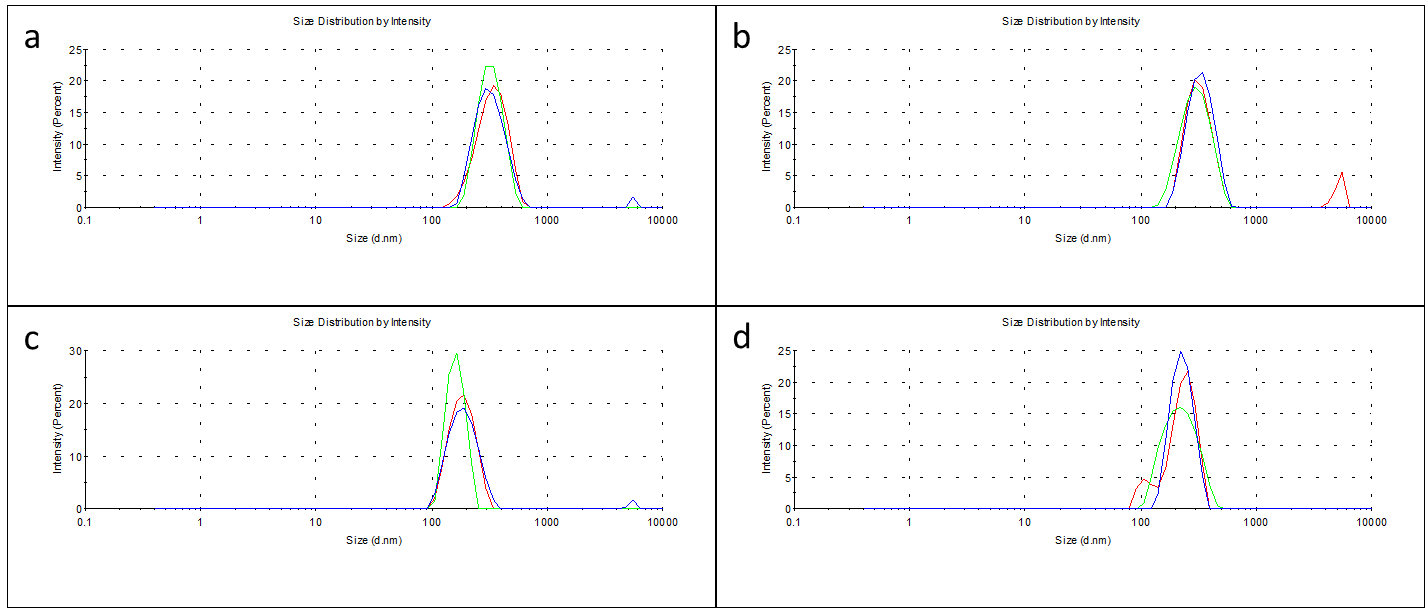
**

**Figure S3.** DLS spectrum. a) G2 in water (50 mg mL^-1^), b) G2d in water (50 mg mL^-1^), c) GNA15 in water (50 mg mL^-1^) and d) GNA15d in water (50 mg mL^-1^).


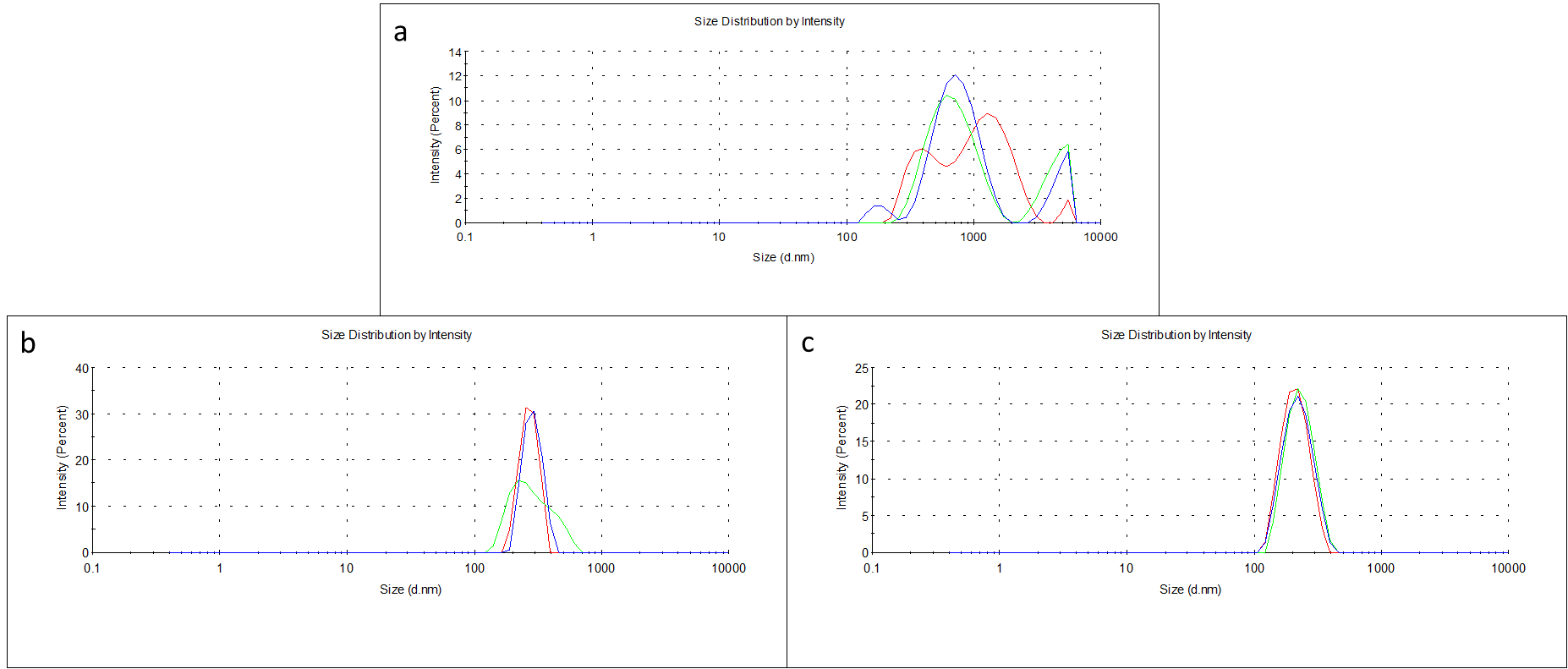


**Figure S4.** DLS spectrum. a) G2d in cell culture media (50 mg mL^-1^), b) GNA15 in cell culture media (50 mg mL^-1^) and c) GNA15d in cell culture media (50 mg mL^-1^).


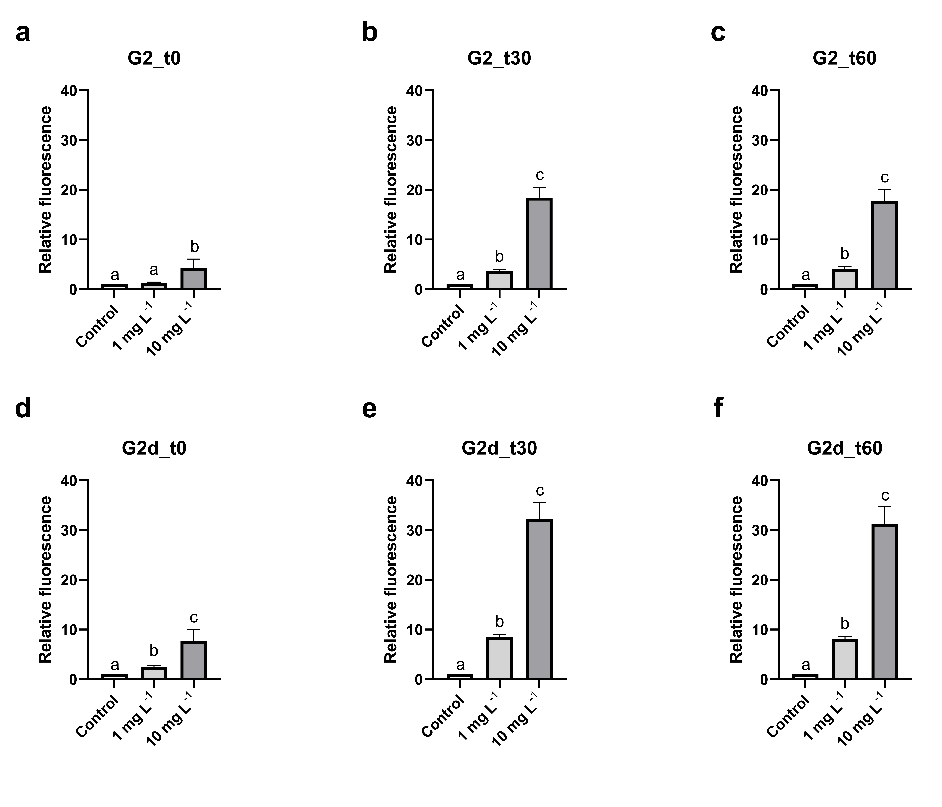


**Figure S5.** A549 ROS response to G2 at t0 (a), t30 (b), and t60 (c); and G2d at t0 (d), t30 (e), and t60 (f). Data represented the mean (± standard deviation, SD) of two independent experiments. Differences were established using a one-way ANOVA followed by multiple comparisons test (Tukey test), and considered significant when *p* ≤ 0.05. Different letters indicate statistically significant differences between treatments. Y axis displays the relative fluorescence fold change in comparison with the control (assigned value of 1), in arbitrary units.


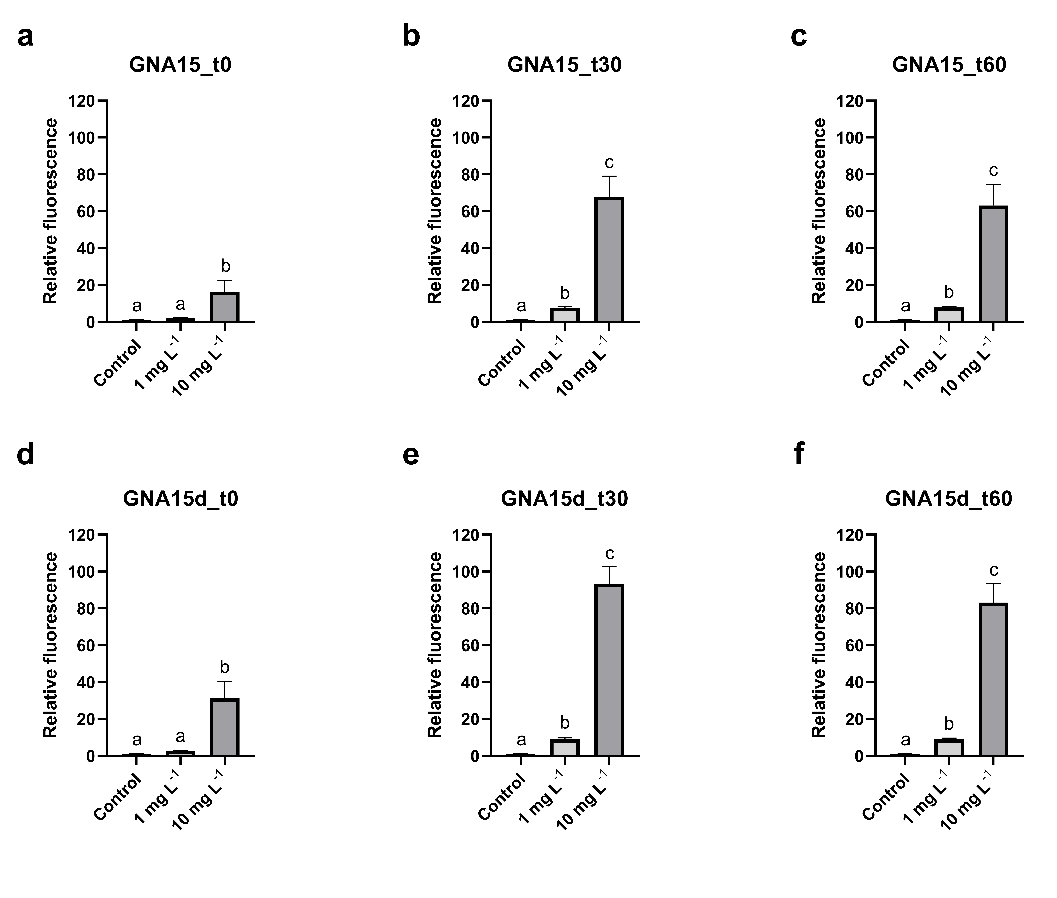


**Figure S6.** A549 ROS response towards GNA15 at t0 (a), t30 (b), and t60 (c); and GNA15d at t0 (d), t30 (e), and t60 (f). Data represented the mean (± standard deviation, SD) of two independent experiments. Differences were established using a one-way ANOVA followed by multiple comparisons test (Tukey test), and considered significant when *p* ≤ 0.05. Different letters indicate significant differences between treatments. Y axis displays the relative fluorescence fold change in comparison with the control (assigned value of 1), in arbitrary units.


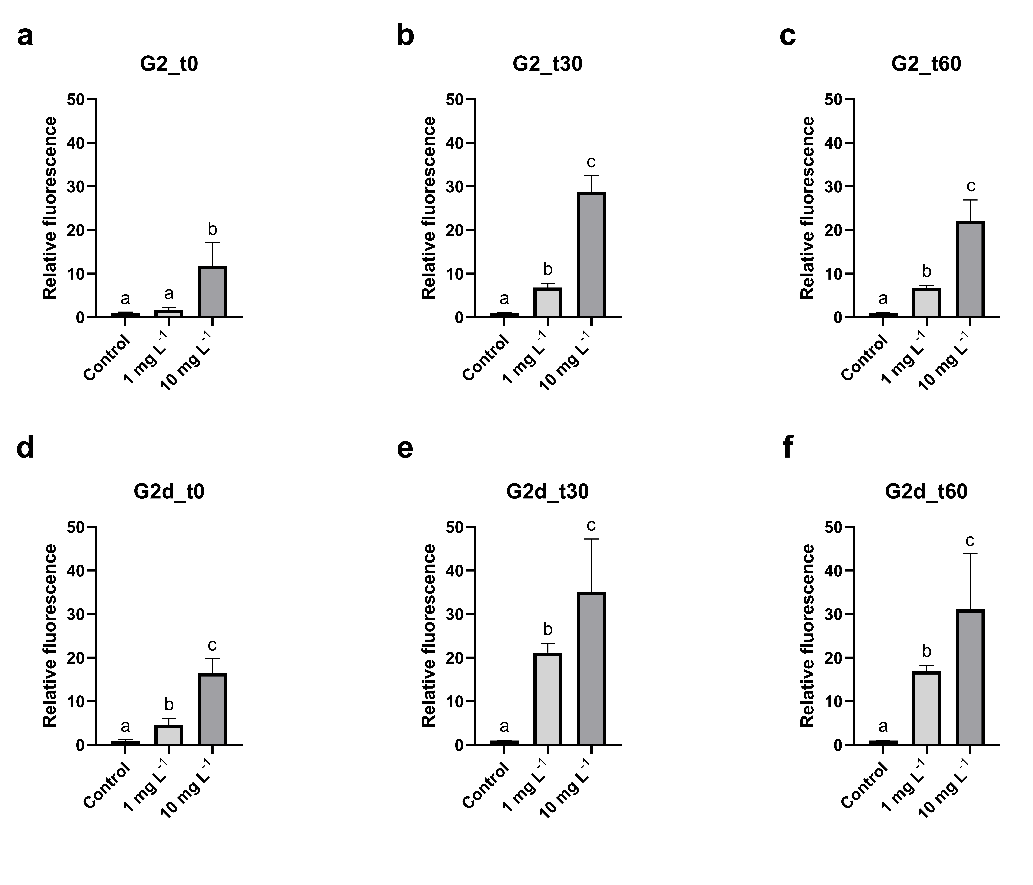


**Figure S7.** HT29 ROS response to G2 GNA15 at t0 (a), t30 (b), and t60 (c); and G2d at t0 (d), t30 (e), and t60 (f). Data represented the mean (± standard deviation, SD) of two independent experiments. Differences were established using a one-way ANOVA followed by multiple comparisons test (Tukey test), and considered significant when *p* ≤ 0.05. Different letters indicate significant differences between treatments. Y axis displays the relative fluorescence fold change in comparison with the control (assigned value of 1), in arbitrary units.


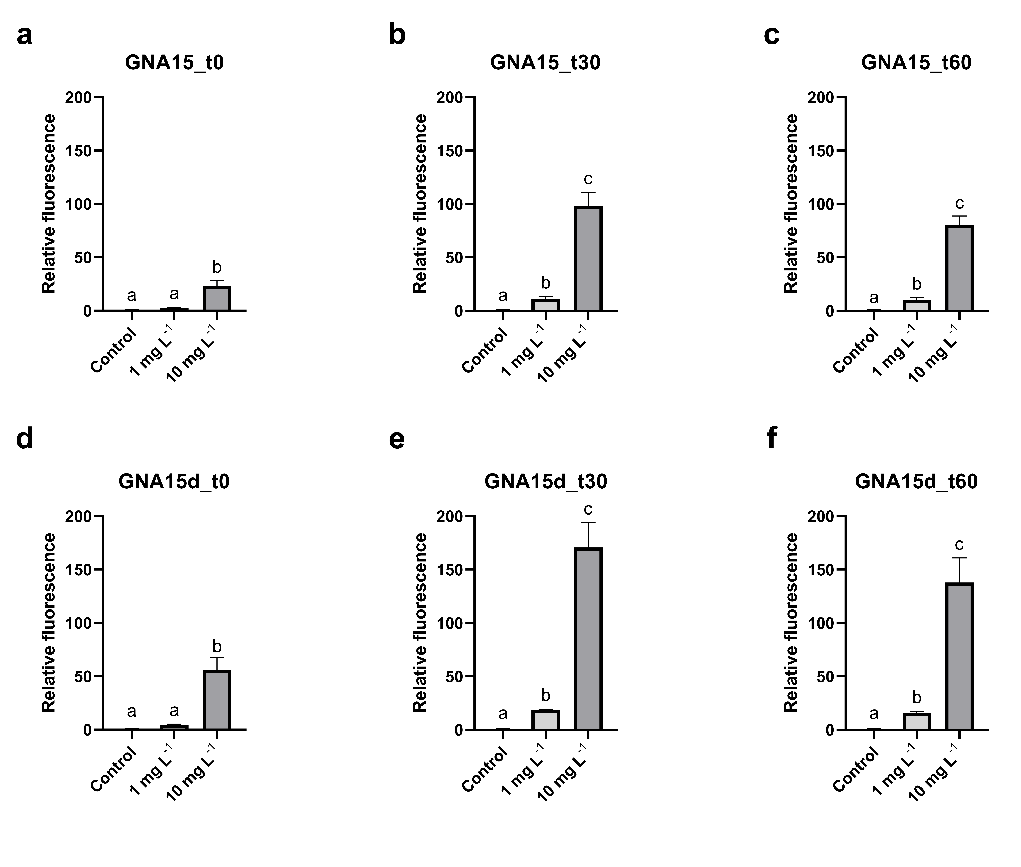


**Figure S8.** HT29 ROS response towards GNA15 at t0 (a), t30 (b), and t60 (c); and GNA15d at t0 (d), t30 (e), and t60 (f). Data represented the mean (± standard deviation, SD) of two independent experiments. Differences were established using a one-way ANOVA followed by multiple comparisons test (Tukey test), and considered significant when *p* ≤ 0.05. Different letters indicate significant differences between treatments. Y axis displays the relative fluorescence fold change in comparison with the control (assigned value of 1), in arbitrary units.





**Figure S9.** EpiDerm^T^ tissues were exposed to different concentrations of G2 (a), G2d (b), GNA15 (c) and GNA15d (d) during 1 h. Tissues treated with PBS were used as negative control. The viability was analysed by MTT assay, and it is expressed as a percent of negative control. Data represented the mean ± standard deviation (SD). Differences were established using a one-way ANOVA followed by multiple comparisons test (Tukey test) and considered significant when *p* ≤ 0.05. The same letter indicates no significant differences between treatments.

**Methods**

The interference of G2 and GNA15 nanomaterials with the MTT to produce formazan was determined under cell-free conditions. The nanomaterial solutions (1, 5 and 10 mg L^-1^) were mixed with MTT (0.5 mg mL^-1^) at a radio 1:1 and incubated at 37 ºC for 3 h. Then, DMSO was added at a ratio 2:1 to the MTT-nanomaterial solutions mixture and incubated for 10 minutes at 37 ºC. The final mixture was centrifuged at 5000 × g for 10 minutes, and the supernatant absorbance was measure at 570 nm in a microplate reader (BioTek Synergy HT). Four replicates were included in the assay. None of the nanomaterials reacted with the MTT reagent in the studied conditions (Fig. S10a).

In addition, we determine in absence of cells the degree of adsorption of the insoluble formazan crystals to the NMs solutions by the incubation of G2 and GNA15 solutions with MTT for 3 h at 37 ºC. After that, the MTT was reduced to formazan by using ascorbic acid, 0.16 mL of the MTT-NM solutions mixture were incubated with 0.066 mL of ascorbic acid (0.05 mM) for 60 minutes at 37 ºC. Then, DMSO was added to MTT- NM solutions-ascorbic acid mixture at a ratio 2:1 and it was incubated for 10 minutes at 37 ºC. The final mixture was centrifuged at 7000 × g for 5 minutes. Finally, the supernatant absorbance was measured at 570 nm with a microplate reader (BioTek Synergy HT). Four replicates were included in the assay. None of the NMs caused a decrease in the measured formazan concentration, suggesting that there was not adsorption of the NMs to the insoluble MTT-formazan crystals (Fig. S10b).


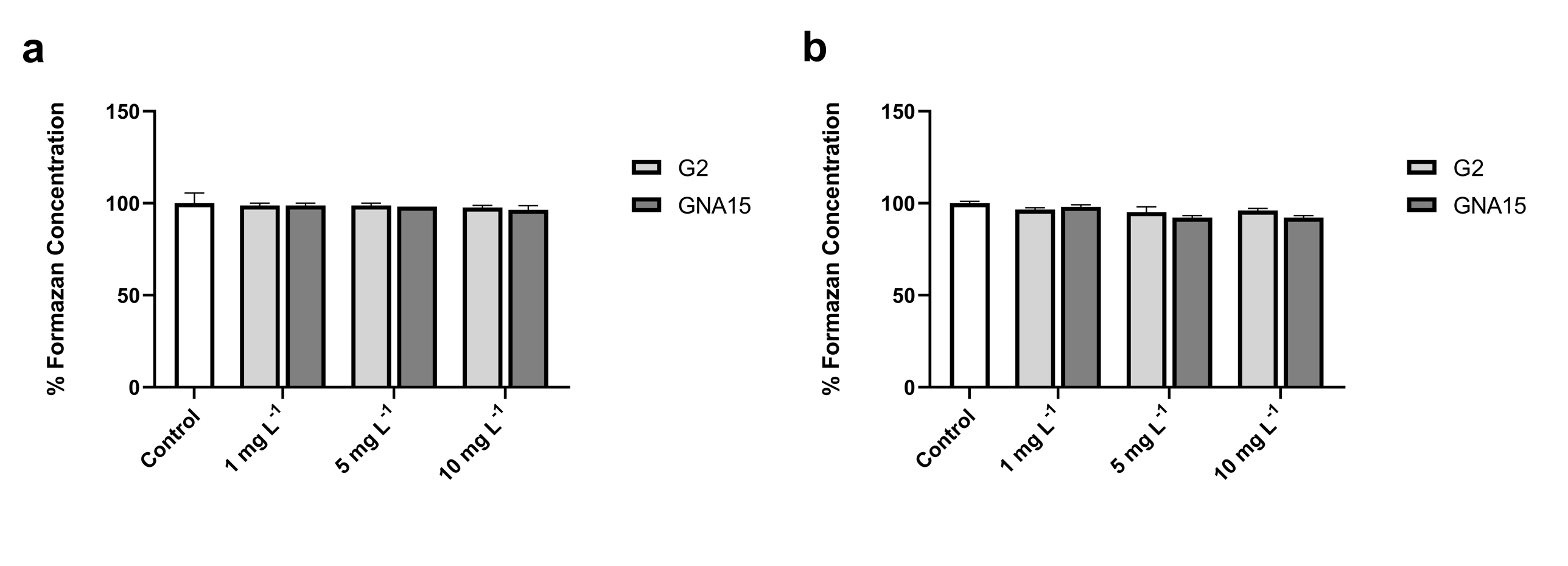


**Figure S10.** Concentration (%) of formazan produced by G2 and GNA15 under cell-free conditions. a) Effect of NM solutions on the generation of formazan through the reduction of MTT reagent. b) Acid ascorbic was added to the NMs solutions to reduce the remaining soluble MTT to formazan. Data represent the mean of 4 replicates (± standard deviation, SD).

The interference of the NMs with optical detection of DCF fluorescence was determined under cell-free conditions and using A549 cells by replacing the assay substrate H_2_DCF-DA by DCF (50 µM). In the cell-free experiments, the NMs solutions (1 and 10 mg L^-1^) were incubated with DFC (50 µM) for 60 minutes at 37 ºC. After that, the fluorescence was measured in a microplate reader (BioTek Synergy HT, excitation wavelength, 485/20; emission wavelength 520/20). Four replicates were included in the assay. All the NMs did not show to cause any interference with the detection of fluorescence DCF at concentrations of 1 and 10 mg L^-1^ (Fig. S11a).

In the cellular assays, cells monolayers were prepared as describe in the Materials and methods section (ROS detection assay in A549 cells) and they were incubated with 100 µL NMs solutions at 1 and 10 mg L^-1^ for 60 minutes at 37 ºC. Then, the cells were washed with HBSS and treated with DCF (50 µM). Fluorescence was measured after 60 minutes in a microplate reader (BioTek Synergy HT, excitation wavelength, 485/20; emission wavelength 520/20). Four replicates were included in the assay. The obtained results indicated that G2 and GNA15 do not interfere in the detection of the DCF fluorescence at concentrations of 1 and 10 mg L^-1^ (Fig. S11b).


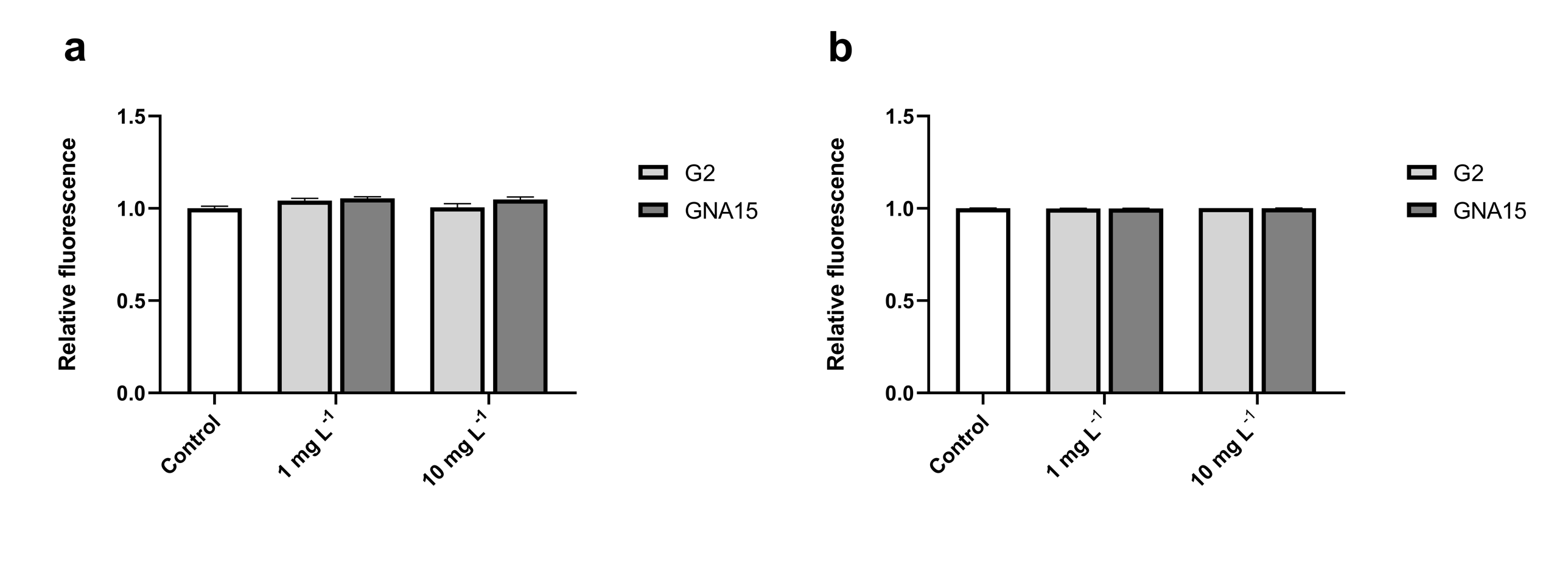


**Figure S11.** a) Interference of G2 and GNA15 with DCF fluorescence under cell-free conditions. b) Interference of G2 and GNA15 with DCF fluorescence using A549 cells. Results are expressed as the relative fluorescence value to the control, which was assigned a value of 1. Data represent the mean of 4 replicates (± standard deviation, SD).

In order to ensure that the NMs do not interfere with the endpoint MTT in the skin irritation test we followed the guideline suggestions. Firstly, we evaluated the intensity of staining of the NMs solutions, and we observed that all the solutions present a high staining, this could indicate that the NMs could stain the tissues. To check the tissue-binding of NMs, we exposed one viable tissue to 30 µL NMs solutions. In parallel, we exposed a tissue to DPBS (negative control). Then, we followed all procedures as described in the guideline, except incubate the tissue for 3 h in culture media without MTT (37 ± 1°C, 5 ± 1 % CO_2_, 90 ± 10 % RH). After 3 h of incubation, we rinsed the tissues and extracted the tissues using 2.0 mL of isopropanol and measured the optical density (OD) at 570 nm.

If the extract from tissues treated with the NMs has an OD between 5 % and 30 % of the negative control tissue (treated with PBS), the NM should be further tested on more tissues using the procedure described above. The real MTT OD (unaffected by interference with the colored test materials) is calculated using following formula:

OD = OD colored tissue (MTT assay) – OD colored tissue (no MTT assay)

If the extract from tissues treated with NMs has an OD < 5 % of the PBS treated control tissue and the tissue viability (determined in MTT assay) is not close to the classification cut-off (50 %), correction of the results is not necessary.

On the other hand, if OD of extract from the tissue treated with NMs is > 30 % of the PBS treated control tissue, additional steps must be performed to determine if the test substance must be considered as incompatible with the test.

Regarding to the studied NMs, they presented an OD < 5 % of the control and they did not reduce the viability 50% of the mean viability of the controls. These results demonstrate that the NMs are compatible with the test.

In addition, we studied the potential of the graphene nanomaterials to interfere with MTT assay. We added 30 µL of the test NM to 1 mL of MTT medium and incubate at 37 ± 1 °C, 5 ± 1 % CO_2_, 90 ± 10 % RH for 1 h. Untreated MTT medium was used as control.

After the incubation, we did not notice that the MTT solution turns blue / purple, which indicates that the test NMs do not reduce MTT.
